# Incorporating indel channels into average-case analysis of seed-chain-extend

**DOI:** 10.1093/bioinformatics/btag312

**Published:** 2026-07-07

**Authors:** Spencer Gibson, Yun William Yu

**Affiliations:** Department of Biomedical Engineering, Carnegie Mellon University, Pittsburgh, PA 15213, United States; Ray and Stephanie Lane Computational Biology Department, Carnegie Mellon University, Pittsburgh, PA 15213, United States

## Abstract

**Motivation:**

Given a sequence $s_{1}$ of *n* letters drawn independently and identically (i.i.d.) from an alphabet of size σ and a mutated substring $s_{2}$ of length m<n, we want to recover the mutation history that generated $s_{2}$ from $s_{1}$. Many modern sequence aligners for this task use seed-chain-extend with *k*-mer seeds. Previously, Shaw and Yu showed linear-gap cost chaining can produce a chain with $1-O\left(\frac{1}{\sqrt{m}}\right)$ recoverability, the proportion of the mutation history that is recovered, in $O\left(mn^{2.43\theta }\;\text{log}\;n\right)$ expected time for seed-chain-extend (assuming pre-seeded reference), where θ<0.206 is the mutation rate under a substitution-only channel and $s_{1}$ is uniformly random. A gap remains between theory and practice, as real genomes include insertions and deletions (indels).

**Results:**

We introduce mathematical machinery to deal with the two new obstacles introduced by indel channels: the dependence of neighbouring anchors and the presence of anchors that are only partially correct. We prove that expected recoverability of an optimal chain is $\geq 1-O\left(\frac{1}{\sqrt{m}}\right)$ and expected runtime is $O(mn^{3.15\cdot \theta_{T}}\;\text{log}\;n)$, given the total mutation rate $\theta_{T}=\theta_{i}+\theta_{d}+\theta_{s}$ (sum of substitution, insertion, and deletion rates) is $\theta_{T}\leq 0.159$. We thus narrow (but not close) the gap between theory and practice.

**Availability and implementation:**

https://github.com/Lazarus42/seed_chainer_indels.

## 1 Introduction

String alignment—i.e. determining the best way to match positions of two similar strings $s_{1}$ and $s_{2}$ under some cost function—has always been one of the central primitives in computational biology, essential for downstream biological analyses like comparing relatedness of genomes or mapping sequenced reads (Koonin *et al.* 2000, Britten 2002, Sirén *et al.* 2021, Nurk *et al.* 2022). It is closely related to the edit distance problem (Chvátal and Sankoff 1975, Ukkonen 1983, Reinert *et al.* 2000, Kiwi *et al.* 2005, Berger *et al.* 2021), as the choice of matching positions in the alignment corresponds to a series of insertions, deletions, and substitutions to transform $s_{2}$ into $s_{1}$. To this end, Needleman-Wunsch (Needleman and Wunsch 1970) and Smith-Waterman (Smith and Waterman 1981) gave dynamic programming exact solutions to the global and local alignment problems in quadratic O(mn) time, where $|s_{1}|=n$ and $|s_{2}|=m$. Although more efficient algorithms exist, e.g. the Four Russians Method has a time complexity of O(mn/ log(n)) (Masek and Paterson 1980), it turns out that in the worst case, we cannot do polynomially better—Backurs and Indyk showed in 2015 that ‘edit distance cannot be computed in strongly subquadratic time (unless SETH is false)’ (Backurs and Indyk 2015).

Of course, there’s more to the story. BLAST (Altschul *et al.* 1990), one of the most highly cited papers of all time (Van Noorden *et al.* 2014), gives a linear-time heuristic for local alignment, at the cost of optimality, and is still to this day one of the primary workhorses of bioinformatics, whereas Smith-Waterman has been relegated to being just a subroutine within heuristic software. More broadly, there are many exact sequence alignment/edit distance algorithms that have subquadratic time complexity under certain conditions–Ukkonen’s method (Ukkonen 1985) for edit distance runs in O(s·min(m,n)) in the worst case, where *s* is the edit distance between the two strings, and Myers’ algorithm (Myers 1986) has $O(m+n+d^{2})$ average-case time complexity where *d* is the minimum edit script between the two strings. Some promising recent work has also led to newer algorithms with provable edit distance guarantees (Ivanov *et al.* 2022, Groot Koerkamp and Ivanov 2024), though they have not yet seen widespread adoption. Other than exact algorithms, there are numerous heuristics that optimize sequence alignment for specific tradeoffs (Chaisson and Tesler 2012, Langmead and Salzberg 2012).

One heuristic of particular interest is ‘seed-chain-extend’, which is used in modern software such as Minimap 2 (Li 2018). The seed-chain-extend heuristic has three stages. In *seeding*, *k*-mer ‘seeds’ are selected on both $s_{1}$ and $s_{2}$, and shared k-mers are marked as ‘anchors’ between the two strings. Afterwards, concordant anchors are *chained* together to form the skeleton of an alignment. Lastly, the space between anchors is filled in using standard worst-case quadratic-time dynamic programming in a process known as *extension*. Seed-chain-extend empirically showcases near quasilinear runtime on the similar genomic strings it is typically applied to, but is not guaranteed to find optimal alignments.

For a long time, bioinformaticians have contented ourselves to this gap between theory and practice. In the last five years, though, theoreticians have made several new breakthroughs by defining a generative model of string evolution and revisiting average-case analysis. Analysis of string algorithms (Lember and Matzinger 2009, Bukh and Hogenson 2020), particularly average-case analysis (Szpankowski 2011), historically made extensive use of generating functions; however, more recent approaches instead used tail-bounds to bound bad events, more akin to the analysis of some randomized probabilistic sketches (Yu and Weber 2022). Ganesh and Sy’s (2020) breakthrough was to show that under their random mutation model, a modified dynamic programming algorithm will compute edit distance in O(n log n) time between a random string $s_{1}$ and a mutated string $s_{2}$ of near equal length (Ganesh and Sy 2020) with high probability. However, their analysis worked because of concordance of their dynamic programming (DP) algorithm with their mutation model, so extending it to more practical but sophisticated heuristics like seed-chain-extend was nontrivial.

In 2023, our research group made substantial progress on proving similar results for seed-chain-extend (Shaw and Yu 2023). Unfortunately, the machinery and techniques we introduced then were insufficiently powerful to address insertions and deletions, so we had to restrict our theoretical results to a substitution-only mutation model. Indels tend to complicate analyses and dependence structures, so most bioinformatics theoreticians either avoid directly working with them (Yu *et al.* 2015, Ondov *et al.* 2016, Edgar 2021, Shaw and Yu 2022), or adjust their algorithm/model to directly capture them (Ganesh and Sy 2020). We were able to run empirical benchmarks with indels that closely tracked our substitution-only theory (including accurate predictions of exponents), so we believed without proof the theorems were also correct for indels (Shaw and Yu 2023). In this sequel, we make progress on narrowing that remaining gap between theory and practice, generalizing our prior machinery and techniques to handle indels.

## 2 Problem introduction and strategy overview

### 2.1 The seed-chain-extend algorithm

Given strings $S,\;S^{\prime }$, we can ask about the likely sequence of edits transforming S into $S^{\prime }$. Seed-chain-extend answers this question with three steps: seeding, chaining, and extension. Seeding is the process by which k-mers (‘seeds’) are selected on both S and $S^{\prime }$ and matching seeds are called anchors. Formally, an anchor of length *k* occurs at (i,j), i.e. starting at position *i* in S and position *j* in $S^{\prime }$, if $S[i:i+k-1]=S^{\prime }[j:j+k-1]$ (indexing is right-inclusive). Second is chaining: a chain $C=((i_{1},j_{1}),\ldots ,(i_{u},j_{u}))$ is a sequence of anchors where $i_{\ell }<i_{\ell +1}$ and $j_{\ell }\leq j_{\ell +1}$ for all 1≤ℓ<u. The chaining score used to determine the optimal chain in this paper is the linear gap cost, where ξ is the gap penalty. For the given chain C, this would be $u-\xi (i_{u}-i_{1}+j_{u}-j_{1})$, or, equivalently, $u-\sum_{\ell =2}^{u}\xi (i_{\ell +1}-i_{\ell }+j_{\ell +1}-j_{\ell })$. Given the optimal chain $C=((i_{1},j_{1}),\ldots ,(i_{u},j_{u}))$, we use classical dynamic programming to extend between consecutive anchors based on any alignment score. In this work, we assume that the reference string S is already seeded, while the query string $S^{\prime }$ is not.

### 2.2 Challenges to analysis of seed-chain-extend

Several difficulties arise in proving average-case results. First, chaining and extension have different optimization objectives. When considering overall performance of the heuristic, it is possible for failures to happen at any stage. By failure, we mean anything that leads to bad downstream events—not finding the correct string alignment or taking too long to find that alignment. A bad chain can result from a failure in chaining, or just a bad selection of anchors in seeding. Similarly, extension failure can be a result of bad chaining, or because the extension procedure does not itself find the right alignment, despite the chaining being ‘good’. Also note that the alignment problem under a mutation model diverges somewhat from the edit distance problem. The best scoring alignment corresponds to some edit distance between the strings, but arguably the ‘correct’ alignment (at least from a biological perspective) is the one that reflects the mutations that happened to transform $s_{1}$ into $s_{2}$.

To resolve these difficulties, in the prequel (Shaw and Yu 2023), we introduced ‘recoverability’, which decoupled chaining accuracy from extension accuracy. Extension is only performed in the gaps between anchors on the chain, so recoverability measures how much of the correct alignment can possibly be recovered given a chain. By structuring our problem thus, we can focus on just the seeding and chaining—i.e. how good is the chain as a starting point for the extension phase. Importantly, recoverability of a chain is a theoretical measure of the goodness of the chain, as opposed to the optimization criterion used to generate the chain, such as linear-gap cost chaining.

### 2.3 Difficulty from indels

Unfortunately, our definition of recoverability in the prequel relied on the substitution-only error model, where the correct alignment of a mutated substring $s_{2}$ to $s_{1}$ is a diagonal in the alignment matrix (the ‘homologous diagonal’). Recoverability was defined as the proportion of the homologous diagonal covered by anchors on the optimal chain and the dynamic programming extension blocks between anchors. With indels, the correct alignment is no longer a straight diagonal. In this sequel, we must generalize the homologous diagonal to a ‘homologous path’.

In the same vein, indels also mess up the matching of indices between $s_{1}$ and $s_{2}$. This is not only notationally very tricky to reconcile, but also make it hard to discuss the dependence structure of positions in anchors, which is necessary for the limited-dependence Chernoff bounds we used in the prequel.

Finally, having indels allows for ‘no operations’ (‘no-ops’)—sequences of mutations that preserve the string—e.g. deletion and insertion of the same letter. This creates a problem with defining recoverability of a homologous path—the alignment specified by the homologous path will have a kink that cannot be found via *k*-mer matching.

The ‘correct’ alignment will not be found by any reasonable alignment method. This is not a problem for recoverability in extension regions, as it still could be an alignment produced by the extension block, but it cannot be found as an anchor, which only gets exact matches. In addition to exact no-ops, where the correct and lowest distance alignments no longer match, indels can also create alternate equivalent edit distance paths, of which only one can be the path history, such as if an insertion duplicates existing letters


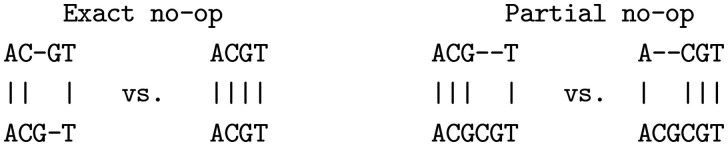


It turns out that this problem cannot be resolved satisfactorily with the prequel’s definition of recoverability, so we will have to introduce a more sophisticated definition of recoverability.

### 2.4 Proof structure and motivation

The basic idea behind the prequel (Shaw and Yu 2023) is that given reasonably low mutation rates, the optimal chain under linear-gap-cost chaining will be close to correct—most of the anchors will be on the homologous diagonal. A gap between anchors can either be homologous if the anchors flanking it are both on the homologous diagonal, or non-homologous if at least one of the two anchors is off the diagonal. Non-homologous gaps can lead to ‘breaks’, which are regions of the string where extension through the gaps does not cover the diagonal, leading to a decrease in recoverability. However, with high probability, each break has size $<\sqrt{m}$ and the number of breaks is small, so the recoverability will be high. Further, the runtime can be bounded by extension time through all the homologous and non-homologous gaps, which are small in an optimal chain.

**Figure 1:**
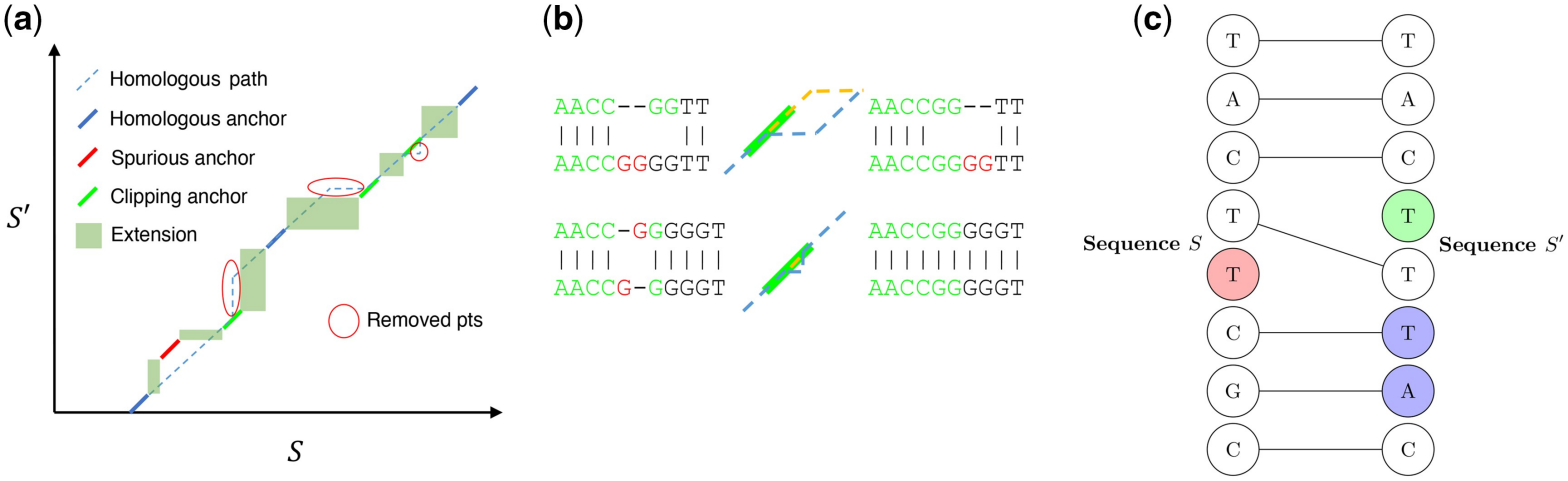
(a and b) Generalized recoverability. (i) Homologous anchors lie entirely on the path, spurious anchors entirely off, and clipping anchors partially on/off. For the purposes of recoverability, we remove points that share an *x*- or *y*-coordinate with a clipped point. (ii) Removing points corresponds to allowing alternate just-as-good paths where the clipping anchor is homologous. (iii) The match graph resulting from the mutation process that gives $S^{\prime }$ from S=TACTTCGC, including a deletion (red), insertion (green), substitutions (blue), and matches (clear). Horizontal lines represent corresponding positions between the sequences. Specifically, in *S*, an insertion of the letter T occurs at position 4, position 5 is deleted and the characters at positions 6, 7, and 8 are mutated.

The intuitive reason the above strategy works is that substitutions are much more likely to break anchors than they are to create spurious anchors. So long as we remain in the regime where sufficiently many anchors can still be found, the optimal chain is close to the correct chain, and the recoverability will be high. When indel channels are added to the mix, the above logic still basically holds. The major difference we handle is a new class of anchors, termed ‘clipping’ anchors (Fig. 1a), which lie partially on the homologous path. Clipping anchors contribute to recoverability in a similar way as homologous anchors: extending through gaps flanked by clipping anchors still contains the bulk majority of the path in the gap. However, they behave like spurious anchors in that the anchors themselves may not recover points on the homologous path.

By generalizing all the theorems in the prequel (Shaw and Yu 2023), we conclude that the expected (prequel) recoverability of an optimal chain is $\geq 1-O\left({\left(\text{log}\;n\right)}^{2}{\left(1-\theta \right)}^{k}\right)$. The reader will find proofs for mathematically involved lemmas and theorems in Appendix D, available as supplementary data at *Bioinformatics* online. Notice that this is asymptotically worse than the $1-O(\frac{1}{\sqrt{m}})$ recoverability in the substitution-only setting, and does not match empirical data, necessitating our new recoverability generalization.

### 2.5 Preliminaries

Three important concepts for our analysis are: the mutation model, which defines how edits are applied to a substring of S to generate $S^{\prime }$, the homologous path, which captures the edit history, and the recoverability of a chain, which measures how ‘close’ the alignment produced by seed-chain-extend is to the true edit history. We also specify the parameter regime under which the theoretical results in this work are guaranteed. Informally, the mutation model consists of making a copy of a substring of S, and, independently, at each position of this copy, there can be a substitution, a deletion, or a random string inserted to the left (or some combination of the three, but always in order of substitution, then deletion, then insertion). The resulting string given by those edits is $S^{\prime }$.

**Definition 1.** [Mutation model] Let $S=x_{1}x_{2}\cdots x_{n}$ be a string where each letter $x_{i}$ is sampled i.i.d. from an alphabet of size σ. The string $S^{\prime }$ is generated by passing a copy of the string $S[p+1:p+m^{\prime }]$ through a mutation channel where for each $1\leq j\leq m^{\prime }$, the copy of S[p+j] undergoes the following mutations independently (although necessarily in this order):

- **Substitution** (probability $\theta_{s}$): The copied symbol is replaced by a different letter, chosen uniformly.
- **Deletion** (probability $\theta_{d}$): The copied symbol is deleted.
- **Insertion** (probability $\theta_{i}$): a random string of length $L∼\text{Geom}(1-\rho_{i}^{\prime })$ is inserted immediately to the left of position j in the copied string. Here, $\rho_{i}^{\prime }$ is the failure probability of the geometric distribution from which insertion lengths are sampled. In this analysis, we bound $\rho_{i}^{\prime }$ away from 0.75, i.e. $0<\rho_{i}^{\prime }<\gamma <0.75$, for any arbitrary γ∈(0,0.75). Intuitively, a larger $\rho_{i}^{\prime }$ leads to longer insertions. We choose γ<0.75 to bound the non-recoverable region sizes later, but everywhere else the analysis only requires γ<1.

Note that to make this a well-defined mutation process these mutations happen in the order listed (substitution, then deletion, then insertion) for each position. Thus, substitution and then deletion leaves the same string as if only deletion were chosen, and it is impossible to delete an insertion. This channel is a slight simplification of the indel channel proposed by Ganesh and Sy (Ganesh and Sy 2020), in that we do not favour runs of consecutive insertions or deletions. Lastly, when we refer to a mutation occurring at position p+j in S, this is shorthand for a mutation occurring at position j in the copy of $S[p+1:p+m^{\prime }]$; the reference string S is never changed.

**Remark 1.** (Mutation channel example). *As a concrete example, consider passing the string S = ACCTACG through the mutation channel described above. At the first position, a substitution (A to T) happens to occur (the copied string is now TCCTACG). At the second position, a deletion occurs and an insertion of CGCG occurs (the copied string is now* T(CGCG)CTACG*). At the next three positions, no mutation occurs (the copied string is still* T(CGCG)CTACG*). At the sixth position, a substitution (C to G) occurs: the copied string is now* T(CGCG)CTAGG*. At the seventh position, no mutation occurs. In this scenario*, S=ACCTACG*and* $S^{\prime }=\mathrm{TCGCGCTAGG}$.

**Remark 2.** *The probability that at position* p+j*, where* $p+1\leq p+j\leq p+m^{\prime }$*, there is no insertion, deletion, or substitution is* $\left(1-\theta_{d})(1-\theta_{s})(1-\theta_{i})=1+(\theta_{i}\theta_{d}+\theta_{i}\theta_{s}+\theta_{d}\theta_{s})-(\theta_{d}+\theta_{s}+\theta_{i})\geq 1-(\theta_{d}+\theta_{s}+\theta_{i}\right)$*. We will refer to the ‘total’ mutation rate as* $\theta_{T}=\theta_{d}+\theta_{s}+\theta_{i}$.

In the definition above, insertion is to the ‘left’ of the position at which it occurs. For example, if S=ACCTAG, and the string *TTACA* is inserted at position 2, then $S^{\prime }$ would be AC(TTACA)CTAG.

If the mutation model only allows for substitutions (Shaw and Yu 2023), the edit history is simple: each position in $S^{\prime }$ is matched with a single corresponding position in S. With this, the optimal alignment was defined simply to be the diagonal path matching every position p+j in S to *j* in $S^{\prime }$, i.e. the set of points $\left\{(p+j,j)|1\leq j\leq m^{\prime }\right\}$.

We extend the same intuition to the indel case: the optimal alignment should capture the edit history transforming S into $S^{\prime }$. We call this edit history the *homologous path*, which generalizes the homologous diagonal from the prequel (Shaw and Yu 2023). One reasonable way to define the edit history is following the canonical alignment from Ganesh and Sy (Ganesh and Sy 2020), which is detailed below.

**Definition 2.** [Homologous path (Inspired by Ganesh and Sy)]. Recall that S is the original length *n* string with i.i.d. letters sampled from the alphabet of size σ, and $S^{\prime }$ is the string resulting from passing a copy of $S[p+1:p+m^{\prime }]$ through the mutation channel where edits E are applied.

We define the homologous path, $P_{H}$, iteratively according to the edits E applied to the copied positions of S. Initially, the homologous path is [(p,0)]. Letting (i,j) be the last element in $P_{H}$, we append points to $P_{H}$ based on the mutations associated with position i+1 in S, assuming $p+1\leq i+1\leq p+m^{\prime }$, following E:

- If no insertion or deletion occurs at position i+1, i.e. a substitution occurs or there is no mutation, append the point (i+1,j+1) to $P_{H}$.
- If an insertion of I letters occurs at position i+1 and no deletion occurs, append points (i,j+1),…,(i,j+I),(i+1,j+I+1) to $P_{H}$.
- If a deletion and no insertion occurs at position i+1, append the point (i+1,j) to $P_{H}$.
- If both an insertion of I letters and a deletion occur at position i+1, append the points (i,j+1),…,(i,j+I),(i+1,j+I) to $P_{H}$.

For example, let *S* = TACTTCGC undergo the following mutations: T is inserted at position 4, position 5 is deleted, the letter at position 6 is substituted to a T, and the letter at position 7 is substituted to an A. These edits result in a string $S^{\prime }$=TACTTTAC. The dependence structure (match graph) between S and $S^{\prime }$ is shown in Fig. 1c, and the homologous path is shown in Fig. A.1, available as supplementary data at *Bioinformatics* online.

**Definition 3.** (Key definitions and assumptions). The constant $\alpha =-{\;\text{log}\;}_{\sigma }(1-\theta_{T})$ represents the expected per-base contribution to the matching length between S and $S^{\prime }$. We will consistently refer to the lengths n:=|S|, $m^{\prime }:=|S[p+1:p+m^{\prime }]|$, and $m:=|S^{\prime }|$. The vocabulary from which letters are sampled to generate S has length σ, and we require that σ≥4.

The length of the seeds, *k*, is given by $k:=C{\;\text{log}\;}_{\sigma }(n)$ for any $C>\frac{3}{1-2\alpha }$. We will write $\text{log}\;:={\;\text{log}\;}_{\sigma }$ for short.

Denote $c_{0}:=\max(\frac{1}{2}\ln(\frac{9}{1+8\gamma }),\frac{21}{\beta })$, where $\beta :={\;\text{log}\;}_{\sigma }(e)$. Recall that γ is the insertion length parameter defined in Definition 1.

The gap penalty constant in the seed-chain-extend linear-gap cost will be $\xi :=\frac{1}{n}$, and $g(n):=\frac{50k}{8{\left(1-\theta_{T}\right)}^{k}}\ln(n)$.

The total mutation rate is given by $\theta_{T}:=\theta_{s}+\theta_{d}+\theta_{i}$, where $\theta_{s},\theta_{d},\theta_{i}$ are the substitution, deletion, and insertion rates in the mutation channel, respectively. We require that $\theta_{T}<0.159$ for the remainder of the theoretical analysis.

**Remark 3** (Relevant bounds on constants and parameters). *Since* σ≥4*and* $\theta_{T}<0.159$*, it follows that* $\alpha <\frac{1}{8}$*, which ensures that* $\frac{3\alpha }{1-2\alpha }<\frac{1}{2}$*. We can write* $C\alpha =\frac{3\alpha }{1-2\alpha }+\delta \alpha$*, from which it follows that* $C\alpha <\frac{1}{2}$*for a sufficiently small* δ>0*. This will be required in subsequent arguments.*

*When working with DNA sequences (* σ=4 *), we can treat* $c_{0}$*as a constant:* $c_{0}=\frac{21}{\beta }\leq 30$*, which will simplify the presentation.*

*The length of the generative region in S is* $m^{\prime }=\Omega (n^{2C\alpha +\epsilon })$*, for an arbitrarily small enough* ϵ>0*. Note such a choice is possible under the described parameter regime since* Cα<1/2*and* $n^{2C\alpha +\epsilon }<n$*. By our choice of variables*, $\sigma^{k}=n^{C}$*and* ${\left(1-\theta_{T}\right)}^{k}=n^{-C\alpha }$*, which we will use repeatedly. Note also that* k=Cβln(n).

*The total mutation rate bound*, $\theta_{T}<0.159$*, was chosen so that* $C\alpha =\frac{3\alpha }{1-2\alpha }+\delta \alpha <\frac{1}{2}$*over all admissible values of* $\theta_{T}$*. This ensures that* $m^{\prime }=\Omega (n^{2C\alpha +\epsilon })\ll n$*. Note that if* $\theta_{T}>0.159$*under this parameter regime, then, in fact*, $m^{\prime }\gg n$*. The constraint* $C>\frac{3}{1-2\alpha }$*ensures that there are no spurious anchors for sufficiently large* n:=|S|*with high probability, as shown in the next section.*

*Parameter regimes and functional relationships were chosen to be realistic while allowing for mathematical convenience. In particular, not all parameter bounds are optimal, and even those that are optimal under the presented mutation model may not be strict for real genomic mutation processes.*

Intuitively, string alignment should recover a minimal edit history consistent with the observed strings under a given scoring function. Excluding ‘no-ops’ from the set of recoverable points ensures that our definition does not penalize such simplicity. For example, if (i,j) is a point in $P_{H}$ and L>0 is maximal such that $S[i+1:i+L]=S^{\prime }[j+1:j+L]$ and $(i+\ell ,j+\ell )\notin P_{H}$ for 1≤ℓ≤L, then any reasonable alignment method will align (i+ℓ,j+ℓ) for 1≤ℓ≤L. Since the edits causing $P_{H}$ to not include these points leaves the substrings of *S* and $S^{\prime }$ unchanged, all points in the offending region are removed when determining recoverability.

**Definition 4.** (Defining non-recoverable regions). For a point $(i,j)\in P_{H}$, define the right non-recoverable region NR(i,j) and the left non-recoverable region NL(i,j) as follows:

First we will define the lengths r(i,j) and l(i,j) of the non-recoverable regions.

$r(i,j)=\max\{t\geq 0|S[i+1:i+t]=S^{\prime }[j+1:j+t]$ and for all $1\leq \ell \leq t,(i+\ell ,j+\ell )\notin P_{H}\}$ if such a t exists, and 0 otherwise.

$l(i,j)=\max\{t\geq 0|S[i-1:i-t]=S^{\prime }[j-1:j-t]$ and for all $1\leq \ell \leq t,(i-\ell ,j-\ell )\notin P_{H}\}$ if such a t exists, and 0 otherwise.

Then the right non-recoverable region is $NR(i,j)=\{(x,y)\in P_{H}|i<x\leq i+r(i,j)\vee j<y\leq j+r(i,j)\}$.

The left non-recoverable region is $NL(i,j)=\{(x,y)\in P_{H}|i\geq x>i-l(i,j)\vee j\geq y>j-l(i,j)\}$.

Having defined the set of non-recoverable points, our generalized recoverability can be defined by removing all non-recoverable points from the homologous path. The recoverability of a chain, $C=((i_{1},j_{1}),\ldots ,(i_{u},j_{u}))$, loosely speaking, is the fraction of the homologous path $P_{H}$, that lies in the extension of anchors in the chain and in gap extensions. We define recoverability to account for all *possible* alignments given by the chain, which means that we count any portion of the homologous path in an extension as ‘recovered’ since, in theory, it could be recovered by some extension algorithm. Recoverability will be the fraction of recoverable points that are, in fact, recovered by the chain, which we formalize below.

**Remark 4.** *Notice that non-recoverable regions occur exactly when either an insertion causes a duplication, when a deletion removes a duplicate region, or both. The chance that this happens for any position (given a random S) is upper bounded by* $\frac{1}{\sigma }(\theta_{d}+\theta_{i})$*The number of points on the homologous path in those non-recoverable regions is then upper bounded by the run of either insertions or deletions which are geometric sums with parameter either* $\left(1-\theta_{d}\right)$*or* $\left(1-\rho_{i}^{\prime }\right)$*. Given that* $\theta_{i}<0.159$*and* γ<0.75*, we can then readily derive a very loose upper bound of* $\frac{4}{\sigma }(\theta_{d}+\theta_{i})m$*points removed from the homologous path. The set of removed points is thus a small constant fraction of* $S^{\prime }$*, created when indels cause alternate alignments with equivalent edit distance as the correct path history.*

**Definition 5.** (Generalized recoverability). Given a chain $C=((i_{1},j_{1}),\ldots ,(i_{u},j_{u}))$, we define the union of all possible alignments for the chain C, Align(C), as:


$$\mathrm{Align}(C)=\overset{u}{\underset{\ell =1}{\cup }}\{(i_{\ell },j_{\ell }),\ldots ,(i_{\ell }+k-1,j_{\ell }+k-1)\}\;\cup \;\overset{u-1}{\underset{\ell =1}{\cup }}Ext(\ell ).$$

Where $\mathrm{Ext}(\ell )=\{i_{\ell }+k-1,\ldots ,i_{\ell +1}\}\times \{j_{\ell }+k-1,\ldots ,j_{\ell +1}\}$. If $i_{\ell }+k-1>i_{\ell +1}$ or $j_{\ell }+k-1>j_{\ell +1}$, then Ext(ℓ)=∅.

Denote the set of non-recoverable points as $U=\underset{(i,j)\in P_{H}}{\cup }(NR(i,j)\cup NL(i,j))$. The recoverability of the chain, R(C), is defined to be:


$$R(C)=\frac{\left|\mathrm{Align}(C)\cap P_{H}\setminus U\right|}{\left|P_{H}\setminus U\right|}$$

Lastly, we introduce the three relevant types of anchors: homologous, clipping, and spurious anchors (Fig. 1a). Visually, anchors always appear as diagonals of length *k* in the alignment matrix. A homologous anchor lies entirely on the homologous path. Unlike the substitution-only case, under indel channels, anchors can touch the homologous path at a number of points without lying entirely on it—these are called clipping anchors. Spurious anchors lie entirely off the homologous path. More formally:

**Definition 6.** (Defining anchor types). For notational ease, let A={(i+t,j+t)∣0≤t≤k−1} and $B=\{(x,y)\in P_{H}|i\leq x\leq i+k-1\;\wedge \;j\leq y\leq j+k-1\}$. If there exists an anchor at (i,j), then A is the set of points belonging to that anchor, and B is the set of points on the homologous path between (inclusive) (i,j) and (i+k−1,j+k−1). The anchor at (i,j) is **homologous** if A=B, **spurious** if A∩B=∅, and **clipping** otherwise.

### 2.6 Anchor-count concentration bounds

The general strategy of our proof is to show that with high probability there are no spurious anchors and that there are not large uncovered gaps in the homologous path before the first anchor or after the last anchor in any optimal chain. In Appendix A, available as supplementary data at *Bioinformatics* online, we analysed the dependency structure between *S* and $S^{\prime }$, which enables us to derive concentration inequalities and tail bounds on the number of spurious anchors $N_{S}$.

In line with the general proof strategy, this section establishes two main results. The first is an expansion-contraction bound on contiguous regions in *S* and $S^{\prime }$: k-mers in $S[p+1:p+m^{\prime }]$ cannot mutate to a string longer than O(k) letters in $S^{\prime }$, and k-mers in $S^{\prime }$ similarly cannot correspond to more than O(k) points in *S*. When the expansion-contraction bound holds, which it will with high probability, the homologous path will be relatively well-behaved. The anchor independence lemmas derived in Appendix A, available as supplementary data at *Bioinformatics* online will then allow us to bound the variance of the number of spurious anchors, $N_{S}$, and to use this bound to show that, with high probability, no spurious anchors occur *at all*.

We proceed with the first result: any *k*-mer in $S[p+1:p+m^{\prime }]$ cannot expand by more than O(k) letters and ℓ-mers where $\ell =\frac{21k}{\beta }$ in $S[p+1:p+m^{\prime }]$ cannot contract to size less than $\frac{(1-\theta_{d})\ell }{2}$ with high probability in the generation of $S^{\prime }$. This choice of ℓ is convenient for the Chernoff bound derived in Lemma B.3 where we show that the probability of any particular ℓ-mer contracting to size $\leq \frac{(1-\theta_{d})\ell }{2}$ is at most $\text{exp}(-\frac{(1-\theta_{d})\ell }{8})$. In order to use a simple union bound over all ℓ-mers and bound all contractions, we need for this probability to be $\leq \frac{1}{n^{2}}$, which is satisfied simply, albeit not tightly, by choosing $\ell =\frac{21k}{\beta }$.

The idea behind this first result’s proof is to show that the probability of expansion or contraction, for each relevant region, is $\leq \frac{1}{n^{2}}$ through Chernoff bounds and moment-generating function inequalities. Applying a union bound over all regions, of which there are at most *n*, gives a probability of $\leq \frac{1}{n}$ that any bad event occurs.

**Lemma 1** (Defining the Expansion-Contraction (EC) space). Let $t_{0}=\frac{1}{2}\ln(\frac{9}{1+8\gamma })$ and $\ell =\frac{21k}{\beta }$. With probability $\geq 1-\frac{2}{n}$, no k-mer in $S[p+1:p+m^{\prime }]$ has more than $\frac{1}{t_{0}}(\frac{2}{\beta }+1)k$ inserted base pairs in $S^{\prime }$ and no ℓ-mer in $S[p+1:p+m^{\prime }]$ contracts to size $\leq \frac{(1-\theta_{d})\ell }{2}$.

**Proof.** Let $E_{1}$ be the event that no *k*-mer in $S[p+1:p+m^{\prime }]$ has more than $\frac{1}{t_{0}}(\frac{2}{\beta }+1)k$ inserted base pairs in $S^{\prime }$ and let $E_{2}$ be the event that no ℓ-mer in $S[p+1:p+m^{\prime }]$ contracts to size $\leq \frac{(1-\theta_{d})\ell }{2}$.

By Lemmas B.2 and B.3, we have $\mathrm{Pr}(E_{1}^{c})\leq \frac{1}{n}$ and $\mathrm{Pr}(E_{2}^{c})\leq \frac{1}{n}$. A simple union bound gives that $\mathrm{Pr}(E_{1}^{c}\vee E_{2}^{c})\leq \frac{2}{n}$, from which it immediately follows that $\mathrm{Pr}(E_{1}\wedge E_{2})\geq 1-\frac{2}{n}$. □

We will refer to the space where the bounded contraction and expansion lemmas are jointly satisfied as EC. Under the *EC* space, we bound the variance of the number of spurious anchors. Using the variance of $N_{S}$, we then bound its value with high probability.

**Lemma 2.** (Working in EC). $E(N_{S}^{2})\leq E{\left(N_{S}\right)}^{2}+2T_{0}k^{2}\frac{mn}{\sigma^{k}}$*where* $T_{0}=2\max({\left(\frac{1}{t_{0}}(\frac{2}{\beta }+1)+3\right)}^{2},4,\frac{21}{\beta })$*. Thus*, $\text{var}(N_{S})\leq T_{0}k^{2}\frac{mn}{\sigma^{k}}$.

The below lemma uses the conditional bounded variance of $N_{S}$ to bound the number of spurious anchors with high probability. From the lemma below, we will conclude that for large enough *n*, there are no spurious anchors with probability $\geq 1-\frac{3}{n}$.

**Lemma 3.** (Working in EC). With probability at least $1-\frac{1}{n}$, the number of spurious anchors is $\leq n^{2-C}+\sqrt{T_{0}}C\;\text{log}(n)n^{\frac{3-C}{2}}.$ Precisely,


$$\mathrm{Pr}\left(N_{S}\geq n^{2-C}+\sqrt{T_{0}}C\;\text{log}(n)n^{\frac{3-C}{2}}|EC\right)\leq \frac{1}{n}$$

**Lemma 4.** (Defining the F1 space). Under EC, for large enough n, there are no spurious anchors with probability $\geq 1-\frac{1}{n}$.

**Proof.** Note that $C>\frac{3}{1-2\alpha }>3$. For large enough *n*, we have that $1>n^{2-C}+\sqrt{T_{0}}C\;\text{log}(n)n^{\frac{3-C}{2}}$. By the previous lemma, $\mathrm{Pr}(N_{S}=0)=\mathrm{Pr}(N_{S}<1)\geq 1-\frac{1}{n}$. □

Similar to how the *EC* space represents the set of events where the expansion-contraction lemma holds, in the *F*1 space there will be no spurious anchors. *F*1 is also a high probability space: $\mathrm{Pr}(F1)\geq \mathrm{Pr}(F1|EC)\mathrm{Pr}(EC)\geq (1-\frac{1}{n})(1-\frac{2}{n})\geq 1-\frac{3}{n}\Rightarrow \mathrm{Pr}(F1)\geq 1-\frac{3}{n}$. In the next section, we show there are no long homologous gaps in the generative region of *S*, and use this fact to bound break lengths with the help of the expansion-contraction lemma.

### 2.7 Bounding homologous gaps

Recall that the general strategy of our proof is to show that with high probability, there are no spurious anchors, and that the vast majority of the path is either covered by (i) a homologous or clipping anchor, or (ii) belongs to an extension region between two anchors. In the previous section, we showed that spurious anchors do not occur with high probability. This section is dedicated to the second portion: proving that there exists some homologous anchor, so that the chain is non-empty, and that they are commonly present along the homologous path with high probability.

A homologous gap is defined to be a region S[p+a,p+b], $1\leq a,b\leq m^{\prime }$ in the generative portion of *S*, for which there are no homologous anchors. We begin by bounding the length of any homologous gap by establishing concentration bounds on homologous anchors in this *k*-dependence case. The lemmas in this section make use of the generative region of *S*, which has length $m^{\prime }$ but the final inequalities are in terms of $|S^{\prime }|=m$. This is fine because the two lengths are equivalent up to a constant ($m^{\prime }=cm$ for a constant c>0) while working under the *EC* space. We will represent fixed constants with variants of *c*; the exact values do not make a difference in the analysis.

We begin with a dependent Chernoff-Hoeffding bound that appears in Yu and Shaw’s paper (Shaw and Yu 2023), originally appearing in Janson’s paper (Janson 2004). The purpose of this lemma is to obtain an exponential (Chernoff) bound for the number of homologous anchors. The lemma makes use of the local dependence of homologous random variables since overlapping k-mers are dependent but non-overlapping k-mers are independent.

**Lemma 5.** (Yu and Shaw) Suppose we have $X=\sum_{a\in A}{\text{Bernoulli}}_{a}(q)$ for some 0<q<1. A proper cover of A is a family of subsets ${\left\{A_{i}\right\}}_{i\in I}$ such that all random variables in $A_{i}\subset A$ are independent and $\underset{i\in I}{\cup }A_{i}=A$. Let χ(A) be the minimum size of the cover, |I|, over all possible proper covers. Then for t≥0,


$$\mathrm{Pr}\left(X\leq EX-t\right)\leq \text{exp}\;\left(-\frac{8t^{2}}{25q|A|\chi (A)}\right).$$

Let $N_{P}$ (‘P’ for preserved, as these regions have no mutations) be the random variable representing the number of *k*-mers in *S* such that there are no mutations in that region. Clearly, each *k*-mer in *S* without mutations is a homologous anchor, i.e. $N_{H}\geq N_{P}$. We can apply the previous theorem to establish a tail bound on the number of homologous anchors with a chain of inequalities: first, we obtain a tail bound for $N_{P}$, and then we bound $N_{H}$ using that $N_{H}\geq N_{P}$.

**Lemma 6.** For any $0\leq t\leq m^{\prime }{\left((1-\theta_{i})(1-\theta_{d})(1-\theta_{s})\right)}^{k}$, we have that $\mathrm{Pr}(N_{H}\leq m^{\prime }{\left((1-\theta_{i})(1-\theta_{d})(1-\theta_{s})\right)}^{k}-t)\leq \text{exp}(-\frac{8t^{2}}{25m^{\prime }k{\left((1-\theta_{i})(1-\theta_{d})(1-\theta_{s})\right)}^{k}})$

**Proof.** We use the previous theorem with $q={\left((1-\theta_{i})(1-\theta_{d})(1-\theta_{s})\right)}^{k}$, the probability that a *k*-mer has no mutation, as shown in Corollary A.2. Recall that $E_{i:i+k-1}$ denotes the random variable taking on the value of 1 if there are no mutations in S[i:i+k−1] and 0 otherwise. Then each set $A_{j}=\{E_{j+tk:j+(t+1)k-1}|t\geq 0\wedge j+tk\leq p+m^{\prime }\}$ contains mutually independent random variables by Corollary A.3. Note $A=\underset{i\in \{1,\ldots ,m^{\prime }\}}{\cup }A_{i}$, and thus the $A_{i}$ form a partition of *A*. This implies χ(A)≤k. Applying the previous theorem yields $\mathrm{Pr}(N_{P}\leq m^{\prime }q-t)\leq \text{exp}(-\frac{8t^{2}}{25m^{\prime }kq})$. Since $N_{H}\geq N_{P}$, we get $\mathrm{Pr}(N_{H}\leq m^{\prime }q-t)\leq \mathrm{Pr}(N_{P}\leq m^{\prime }q-t)$. Result follows. ▪

We can apply the previous lemma to obtain a Chernoff bound for the probability of a gap of length ℓ occurring. It will follow there is no ‘large’ homologous gap with probability $\geq 1-\frac{1}{n}$.

**Lemma 7.** For any interval of length ℓ in $S[p+1:p+m^{\prime }]$, the probability that no homologous anchor occurs is


$$\leq \text{exp}\;\left(-\frac{8\ell {\left(1-\theta_{T}\right)}^{k}}{25k}\right).$$

**Proof. **Each interval of length ℓ in $S[p+1:p+m^{\prime }]$ can be viewed as an identically distributed length ℓ version of it. Applying the previous lemma with $m^{\prime }=\ell$ and $t=\ell {\left(1-\theta_{T}\right)}^{k}$ gives $\text{Pr}(N_{H}\leq 0)=\text{Pr}(N_{H}=0)\leq \text{exp}(-\frac{8\ell {\left((1-\theta_{i})(1-\theta_{d})(1-\theta_{s})\right)}^{k}}{25k})\leq \text{exp}(-\frac{8\ell {\left(1-\theta_{T}\right)}^{k}}{25k})$ since ${\left((1-\theta_{i})(1-\theta_{d})(1-\theta_{s})\right)}^{k}\geq {\left(1-\theta_{T}\right)}^{k}$. ▪

Using the previous lemma, we show that every region of length $g(n)=\frac{C\cdot 50}{8}\text{log}(n)\ln(n)n^{C\alpha }$ contains a homologous anchor. This lemma establishes that homologous anchors are ‘dense’. Denote the space under which there are no homologous gaps of size ≥g(n) by *F*2: we will be in the space *F*2 with probability $\geq 1-\frac{1}{n}$.

**Lemma 8.** (Defining the F2 space). With probability $\geq 1-\frac{1}{n}$, no homologous gap in $S[p+1:p+m^{\prime }]$ has size greater than


$$g(n)=\frac{50k}{8{\left(1-\theta_{T}\right)}^{k}}\ln(n)=\frac{C\cdot 50}{8}\text{log}(n)\ln(n)\cdot n^{C\alpha }$$

plus a small C log n term we ignore because it is small asymptotically.

**Proof.** The proof is identical to Lemma 6 from Yu and Shaw (Shaw and Yu 2023). We will replicate the proof for this crucial lemma.

Let $\ell =g(n)=\frac{50k\ln(n)}{8{\left(1-\theta_{T}\right)}^{k}}$. Define ${\text{HG}}_{1},\ldots ,{\text{HG}}_{m^{\prime }-\ell +1}$ be the random variables indicating if there is a homologous gap of length ℓ at a given position, i.e. ${\text{HG}}_{i}=1$ when no *k*-mer in S[p+j:p+j+ℓ−1] is part of a homologous anchor. Note that $E[\sum_{i=1}^{m^{\prime }-\ell +1}HG_{i}]\leq \frac{m^{\prime }}{n^{2}}\leq \frac{1}{n}$. Applying Markov’s inequality, we get that $\text{Pr}(\sum_{i=1}^{m^{\prime }-\ell +1}{\text{HG}}_{i}\geq 1)=\text{Pr}(\sum_{i=1}^{m^{\prime }-\ell +1}{\text{HG}}_{i}\geq n\cdot 1/n)\leq \frac{1}{n}$, and the result follows. ▪

The previous lemma shows that within the high probability F2 space, there are no homologous gaps in $S[p+1:p+m^{\prime }]$ of size greater than O(g(n)); homologous anchors are ‘dense’ under *F*2. In the following lemma, we work under the intersection G=F1∧EC∧F2 to show that there are at most O(g(n)) unrecovered points before the first anchor or after the last anchor of any optimal chain.

**Lemma 9.** (Working in G). For any optimal chain $C=((i_{1},j_{1}),\ldots ,(i_{u},j_{u}))$, there are at most O(g(n)) unrecovered points on $P_{H}$ before the first anchor of C or after the last anchor. Formally, let $S=\{(x,y)\in P_{H}|(x\leq i_{1}\vee y\leq j_{1})\vee (x\geq i_{u}\vee y\geq j_{u})\}$. Then |S|=O(g(n)).

**Proof.** Consider the first anchor $\left(i_{1},j_{1}\right)$. Working in G, there are no spurious anchors, so $\left(i_{1},j_{1}\right)$ must be a homologous anchor or a clipping anchor. In either case, there is a point on the anchor that belongs to the homologous path. Call this point (i,f(i)). Under *F*2, there are no homologous gaps of size ≥g(n), so there is some homologous anchor occurring at $i_{0}<i_{1}$, A(i,f(i))=1, such that $i-i_{0}\leq g(n)$. Under *EC*, since $i-i_{0}\leq g(n)$, there cannot be more than $\frac{1}{t_{0}}(\frac{2}{\beta }+1)g(n)$ insertions between $f(i_{0})$ and f(i). Thus, $f(i)-f(i_{0})\leq 2\frac{1}{t_{0}}(\frac{2}{\beta }+1)g(n)$. It follows that $\left(i-i_{0})+(f(i)-f(i_{0}))\leq 3\frac{1}{t_{0}}(\frac{2}{\beta }+1)g(n\right)$. Adding this homologous anchor to the chain changes its score by at least $1-\xi (i-i_{0}+f(i)-f(i_{0}))$, which is greater than 0, since $\xi =\frac{1}{n}$ and n≫g(n). Thus, adding it to the chain increases its score. It follows that the first anchor $\left(i_{1},j_{1}\right)$ in any optimal chain has some point on it at most g(n) away from the start of the generative position, p+1. We once again have that the *y*-value of this point can be at most $2\frac{1}{t_{0}}(\frac{2}{\beta }+1)g(n)$ larger than the first point on the homologous path. Thus, at most $g(n)+2\frac{1}{t_{0}}(\frac{2}{\beta }+1)g(n)\leq 3\frac{1}{t_{0}}(\frac{2}{\beta }+1)g(n)$ points are missed before this first anchor. The same logic shows that there can be at most $3\frac{1}{t_{0}}(\frac{2}{\beta }+1)g(n)$ points missed on the homologous path after the last anchor. Combining these facts, at most $6\frac{1}{t_{0}}(\frac{2}{\beta }+1)g(n)=O(g(n))$ points are missed before or after any optimal chain. ▪

## 3 Recoverability and runtime theorems

Recall the three high probability spaces we defined **(Informal)**:

-
[F1] , defined in Lemma 4: For large enough *n*, there are no spurious anchors at all with probability $\geq 1-\frac{3}{n}$.
-
[EC] , defined in Lemma 1: Each Θ(k)-mer in *S* corresponds to an Θ(k)-mer in $S^{\prime }$ after edits with prob. $\geq 1-\frac{2}{n}$.
-
[F2] , defined in Lemma 8: With probability $\geq 1-\frac{1}{n}$, no homologous gap in $S[p+1:p+m^{\prime }]$ has size greater than $O(g(n))=O(\frac{C\cdot 50}{8}\text{log}(n)\ln(n)\cdot n^{C\alpha })$.

As before, we will analyse seed-chain-extend under the intersection of these spaces, G=F1∧F2∧EC. Recall again that *G* is itself a high probability space: $\mathrm{Pr}(G^{c})\leq \mathrm{Pr}(F1^{c})+\mathrm{Pr}(F2^{c})+\mathrm{Pr}(EC^{c})\leq \frac{6}{n}\Rightarrow \mathrm{Pr}(G)\geq 1-\frac{6}{n}$.

We now move on to the first main result: proving the expected recoverability of seed-chain-extend with indels is $\geq 1-O\left(\frac{1}{\sqrt{m}}\right)$ for large enough *n*. To this end, we lower bound the recoverability of any chain while working in *G*.

**Lemma 10** (Working in G).

Let $C=((i_{1},j_{1}),\ldots ,(i_{u},j_{u}))$ be an optimal chain. Let the first and last points of the homologous path be $\left(i_{s},j_{s}),(i_{e},j_{e}\right)$, respectively. Then the recoverability of C is


$$R(C)\geq 1-\frac{\left(i_{1}-i_{s})+(j_{1}-j_{s})+(i_{e}-i_{u})+(j_{e}-j_{u}\right)}{\left|P_{H}\right|}.$$

**Proof. **Any point $(x,y)\in P_{H}$ for which $0\leq x<i_{1}$ or $0\leq y<j_{1}$ or $x\geq i_{u}+k$ or $y\geq j_{u}+k$ is not recoverable. There are at most $i_{1}+j_{1}+(i_{e}-i_{u})+(j_{e}-j_{u})$ such points. Consider any point $(x,y)\in P_{H}$ such that $i_{1}\leq x\leq i_{u}$ and $j_{1}\leq y\leq j_{u}$. These points belong to three categories:

1. **Anchor Points**. Since (x,y) lies on an anchor, it is recovered.
2. **Between anchors**. There exist anchors $\left(i_{p},j_{p}\right)$ and $\left(i_{p+1},j_{p+1}\right)$ such that $i_{p}+k-1\leq x\leq i_{p+1}$ and $j_{p}+k-1\leq y\leq j_{p+1}$. Then (x,y) lies in an extension box and is recovered.
3. **Overlap with clipping anchors**. (x,y) belongs to a region covered by a clipping anchor. Specifically, there exists a clipping anchor $\left(i_{\ell },j_{\ell }\right)$ such that $i_{\ell }\leq x\leq i_{\ell }+k-1\vee j_{\ell }\leq y\leq j_{\ell }+k-1$. Since (x,y) is not recovered, it does not lie on the clipping anchor itself. Thus, it belongs to a non-recoverable region, which is covered by *U*.

Thus, each point contained within the bounds of the chain is either recovered or part of an unrecoverable region, in which case it is contained in *U*, and the result follows. ▪

**Theorem 1** (Recoverability theorem). *The expected recoverability of an optimal chain*, $C=((i_{1},j_{1}),\ldots ,(i_{u},j_{u}))$*, is* $\geq 1-O\left(\frac{1}{\sqrt{m}}\right)$*for large enough n.*

**Proof.** Recall that the recoverability of a chain C is defined as $R(C)=\frac{\left|\mathrm{Align}(C)\cap P_{H}\setminus U\right|}{\left|P_{H}\setminus U\right|}$. We showed this is $\geq 1-\frac{\left(i_{1}-i_{s})+(j_{1}-j_{s})+(i_{e}-i_{u})+(j_{e}-j_{u}\right)}{\left|P_{H}\right|}$, in G=F1∧F2∧EC in the previous lemma. Note that $\mathrm{Pr}(G)\geq 1-\frac{2}{n}-\frac{3}{n}-\frac{1}{n}=1-\frac{6}{n}$.

Working in *G*: by Lemma 9, the number of points missed before the start of the chain or after the end of the chain is $\leq (i_{1}-i_{s})+(j_{1}-j_{s})+(i_{e}-i_{u})+(j_{e}-j_{u})\leq 6\frac{1}{t_{0}}(\frac{2}{\beta }+1)g(n)$. Recall that $g(n)=\frac{C\cdot 50}{8}\text{log}(n)\ln(n)n^{C\alpha }$. For large enough *n*, $n^{C\alpha }<\sqrt{m}$, so $\frac{g(n)}{m}\leq O(\frac{1}{\sqrt{m}})$. Thus, $E(\frac{\left(i_{1}-i_{s})+(j_{1}-j_{s})+(i_{e}-i_{u})+(j_{e}-j_{u}\right)}{\left|P_{H}\right|}|G)=O(\frac{g(n)}{m})=O(\frac{1}{\sqrt{m}})$. Combining it all, $E(R)\geq E(R|G)\mathrm{Pr}(G)=\left(1-O\left(\frac{1}{\sqrt{m}}\right)\right)(1-6/n)=1-O\left(\frac{1}{\sqrt{m}}\right).$ ▪

Finally, we prove that the runtime of seed-chain-extend is $O(mn^{C\alpha }\;\text{log}\;n)$ in expectation, given that the reference string *S* is already seeded. This follows by establishing two results: (1) the chaining runtime is $O(mn^{C\alpha }\;\text{log}\;n)$ since $E[N\;\text{log}\;N]=O(mn^{C\alpha }\;\text{log}\;n)$, where *N* is the number of anchors between $S,S^{\prime }$; (2) the extension runtime between anchors in an optimal chain is upper bounded by $O(mn^{C\alpha }\;\text{log}\;n)$. Since the runtime of seed-chain-extend is dominated by chaining with extension, this proves the result. We show the second point by proving extension runtime through any optimal chain is at most the extension runtime through a related chain containing only homologous anchors with no mutations (Appendix C, available as supplementary data at *Bioinformatics* online).

**Theorem 2** (Runtime theorem). *Given that the reference string S is already seeded, the expected runtime* $E[T_{\mathrm{SCE}}]$*of seed-chain-extend is* $O(mn^{C\alpha }\;\text{log}\;n)$.

**Proof.** The runtime $T_{\mathrm{SCE}}$ of any chain C is the runtime of seeding the query $S^{\prime }$, chaining, and extension. Seeding $S^{\prime }$ is fast: it takes O(m) time. We have that $T_{\mathrm{SCE}}=O(m)+T_{\mathrm{Chain}}+T_{\mathrm{Ext}}$. By Lemma C.1, $E[T_{\mathrm{Chain}}]=O(mn^{C\alpha }\;\text{log}\;n)$, and by Lemma C.5, $E[T_{\mathrm{Ext}}]=O(mn^{C\alpha }\;\text{log}\;n)$. Thus, $E[T_{\mathrm{SCE}}]=O(m)+O(E[T_{\mathrm{Chain}}])+O(E[T_{\mathrm{Ext}}])=O(mn^{C\alpha }\;\text{log}\;n)$. ▪

## 4 Experimental results

We set $n=\left\lfloor 4^{\frac{k}{C}}\right\rfloor +10$ (from $k=C{\;\text{log}\;}_{4}(n)$, the additional +10 ensures that $m^{\prime }<n$), $C=\frac{3}{1-2\alpha }$ (recall that $\alpha =-{\;\text{log}\;}_{\sigma }(1-\theta_{T})$), and $m^{\prime }=n^{\frac{2C\alpha +1}{2}}$, so that $m^{\prime }=\Omega (n^{2C\alpha +\epsilon })\ll n$. The recoverability experiments are run with k∈[20,44], and the runtime experiments use k∈[26,44]. These ranges were selected to capture asymptotic behaviour rather than finite-size effects.

For each choice of parameters $k,n,m^{\prime },\theta_{T},$ and γ (recall that γ governs the average insertion string length), we perform 100 iterations of generating the reference string *S* by sampling letters i.i.d. from {A,C,T,G}, passing a substring of *S* with length $m^{\prime }$ through the mutation channel detailed in Definition 1 to generate $S^{\prime }$, and running seed-chain-extend on *S* and $S^{\prime }$. The individual mutation rates, $\theta_{s},\theta_{d},\theta_{i}$ are randomly chosen s.t. $\theta_{T}=\theta_{s}+\theta_{d}+\theta_{i}$.

For each choice of parameters, we record the average recoverability and length of $S^{\prime }$ over 100 trials. We then fit a linear regression on $\text{log}(1-\hat{E}(R))$ against $\text{log}\;\hat{E}(|S^{\prime }|)$; the slope of the best fit line is the scaling exponent. In particular, if $\text{log}(1-\hat{E}(R))\approx a+b\;\text{log}\;\hat{E}(|S^{\prime }|)$, then $1-\hat{E}(R)\approx c{\left(\hat{E}(|S^{\prime }|)\right)}^{b}$, from which we conclude that $\hat{E}(R)\approx 1-O{\left(\hat{E}(m)\right)}^{b})$, where $m:=|S^{\prime }|$. The average length of $S^{\prime }$ is used in the regression since it is stochastic across trials and observing repeated instances of any fixed length is infeasible. Under *EC*, $\left|S^{\prime }\right|$ and |S| are interchangeable up to a constant, which is absorbed into the intercept in the log-log fit and does not affect the slope.

**Figure 2:**
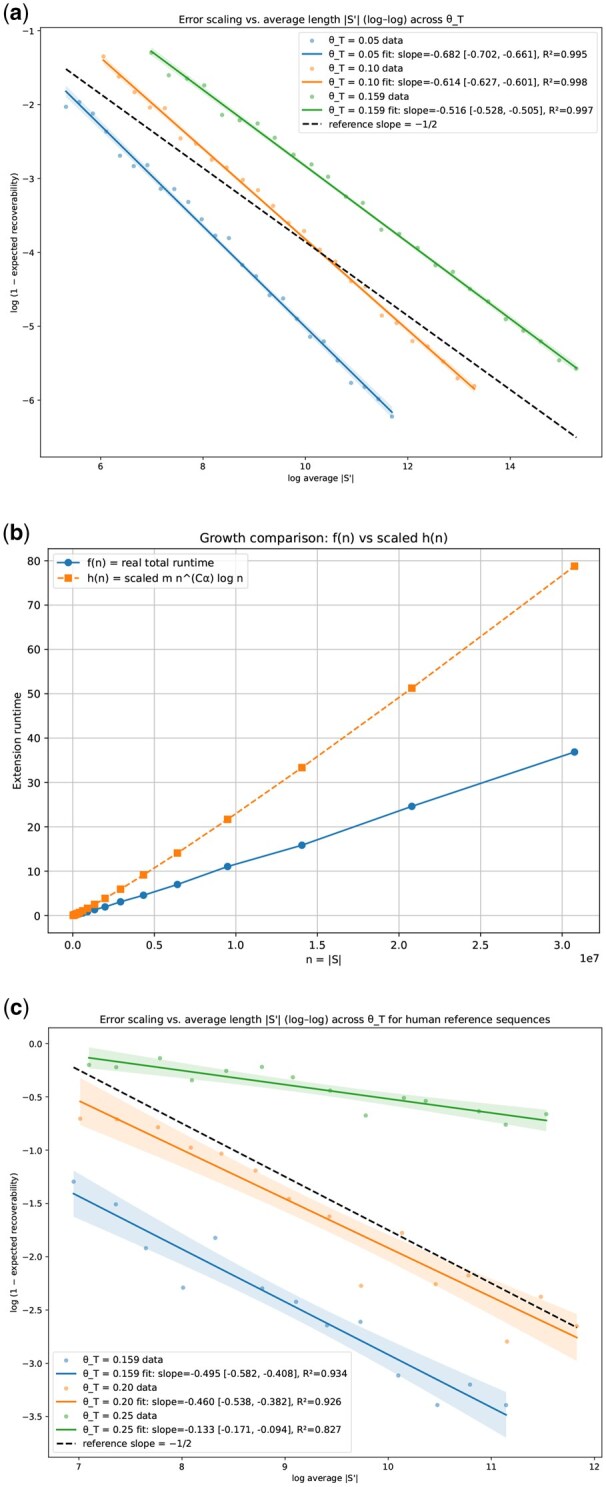
Recoverability analyses across mutation parameters and corresponding runtime scaling behaviour. (a) Log-log fit of average recoverability error versus average length of $S^{\prime }$ for $\theta_{T}\in \{0.05,0.10,0.159\}$. (b) The average empirical runtime (blue, the flatter curve) and the scaled theoretical prediction $O(mn^{C\alpha }\;\text{log}\;n)$ (orange, the steeper curve) for 26≤k≤44, γ=0.5, and $\theta_{T}=0.10$. The growth of the empirical average runtime is consistent with $E(T_{\mathrm{SCE}})=O(mn^{C\alpha }\;\text{log}\;n)$. (c) The log-log plot of average recoverability error against the average length of $S^{\prime }$ for $\theta_{T}\in \{0.159,0.20,0.25\}$. Here, *S* is from human chromosome 1 of the GRCh38 (hg38) reference genome.

Figure 2a shows the rate of convergence of $1-\hat{E}(R)$ to 0 throughout the parameter regime $\theta_{T}\leq 0.159$ and γ=0.5. The experimental results indicate that the rate of convergence is at least as quick as $\frac{1}{\sqrt{m}}$ (recall that $m:=|S^{\prime }|$) for each value of $\theta_{T}\in \{0.05,0.10,0.159\}$. This supports the theoretical recoverability result shown in Theorem 1, that $E(R)=1-O(\frac{1}{\sqrt{m}})$ for all $\theta_{T}\leq 0.159$.

The experimental runtime results are presented in Fig. 2b. Here, we fix γ=0.5, and $\theta_{T}=0.10$. Similar to the recoverability experiments, *S* and $S^{\prime }$ are generated 100 times for each k∈[26,44], and the average total runtime (chaining + extension) and average predicted runtime are recorded. The real average runtime is denoted by f(n), since *n* is the only unfixed parameter in this experiment, and h(n) is the predicted runtime scaled by a factor of $2\times \frac{\text{Measured}\;\text{runtime}\;\text{at}\;n=26}{\text{Predicted}\;\text{runtime}\;\text{at}\;n=26}=3.87\times 10^{-8}$ to obtain a common reference point. Figure 2b empirically supports that the expected total runtime of seed-chain-extend is $O(mn^{C\alpha }\;\text{log}\;n)$.

To stress-test the theoretical recoverability results, we run seed-chain-extend on sequences *S* of length $n=\left\lfloor 4^{\frac{k}{C}}\right\rfloor +10$ randomly sampled from contiguous regions of human chromosome 1 of the GRCh38 (hg38) reference genome (Schneider *et al.* 2017). We use k∈[20,33], $C=\frac{3}{1-2\alpha }$, γ=0.5, and α=−log(1−0.159)≈0.1249, corresponding to their limit values under the assumptions and mutation model described in Section 2.5. We vary $\theta_{T}\in \{0.159,0.20,0.25\}$, and for each $\theta_{T}$, we record the average recoverability of seed-chain-extend and the average length of the generated $S^{\prime }$ over 10 trials. Note that the number of trials and the range of *k* are smaller in this experiment due to the substantially larger computational burden. Figure 2c presents the results of this experiment. As $\theta_{T}$ increases to 0.20, the convergence rate of the average recoverability error slightly degrades. When $\theta_{T}=0.25$, $E(R)\approx 1-O(m^{-0.139})$, which has substantially worse convergence to 1 than the $\frac{1}{\sqrt{m}}$ convergence guaranteed under the parameter regime used throughout the paper.

In Appendix Section J, available as supplementary data at *Bioinformatics* online, we present further experimental results measuring the empirical recoverability convergence rate and runtime under regimes that differ substantially from the one presented here. In the first experiment, we take *S* as a substring of human chromosome 1 (hg38) and use INDELible (Fletcher and Yang 2009) to apply realistic (non-i.i.d., symmetric power-law indel length distributions) mutations to it; in the second, we use PBSIM3 (Ono *et al.* 2022) to introduce realistic long-read sequencing errors into a noisy copy ($S^{\prime }$) of the reference string *S*.

## 5 Limitations

Although indel modelling is more realistic than purely considering substitutions, as the prequel (Shaw and Yu 2023) did, we still assume that the genomes are random strings. Thus, our guarantees fail precisely in repetitive regions of the genome, especially for larger scale duplications. Of course, large-scale duplications empirically cause substantial problems for read mappers, so this is in part a limitation of seed-chain-extend as a method, rather than failure of our theory, but it still is a remaining gap between theory and practice.

Furthermore, genomic mutations are often correlated, violating both independent and geometric length of insert assumptions. We also have a slightly asymmetric mutation model, whereby deletions are independent at every position but insertions come in runs, whereas in biology, it seems that deletions often also come in runs.

## 6 Conclusion

Under moderate assumptions, expected recoverability of an optimal chain is $\geq 1-O(\frac{1}{\sqrt{m}})$ and expected runtime is $O(mn^{C\alpha }\;\text{log}\;n)\leq O(mn^{3.15\cdot \theta_{T}}\;\text{log}\;n)$, for a mutation model with indels and substitutions. This extends the paper of Yu and Shaw (Shaw and Yu 2023), which addressed the substitution-only regime. Our main technical contributions are the introduction of *clipping* anchors, which lie partially on the homologous path, the application of concentration inequalities to bound the probability of unfavourable edit histories, and a more complete understanding of why the seed-chain-extend heuristic is successful, further narrowing the theory-practice gap.

## Supplementary Material

btag312_Supplementary_Data

## Data Availability

Select experiments described in this article use data from the Genome Reference Consortium Human Build 38 (GRCh38). No new primary data were generated in support of this research.
