# Supplementary material for: Incorporating indel channels into average-case analysis of seed-chain-extend
Source: Bioinformatics. 2026 Jul 7;42(Suppl 1):btag312. doi: 10.1093/bioinformatics/btag312 (PMC13340171; doi:10.1093/bioinformatics/btag312)
Supplement: btag312_Supplementary_Data [file btag312_supplementary_data.pdf]

Appendix to Incorporating indel channels into average-case analysis of seed-chain-extend

Spencer Gibson and Yun William Yu

A. Dependence between S and S'

Tools

In this section, we present two tools that simplify our analysis: match variables, which are the random variables for the event that a particular letter on S matches some letter on S', and a function that tracks where non-deleted positions on S map to on S'. Since our analysis deals with various types of anchors, we formally define each of them in this section.

To begin, we define the concept of a match variable. As stated before, these variables are indicator random variables for the event that positions on S and S' share the same letter. We can then define the random variable for the event that an anchor occurs at a position tuple of S and S'.

Definition A.1 Let $M(i,j) = \mathbf{1}\{S[i] = S'[j]\}$ be the indicator random variable detecting if S and S' share the same character at positions i and j. Define $A(i,j) = \prod_{\ell=0}^{k-1} M(i+\ell, j+\ell)$, the indicator random variable for an anchor occurring at (i,j).

Let $x \in [p+1, p+m']$ be an index in the generative region of S. If the character at position x is not deleted in the mutation process, then there is a unique corresponding position in S'. One useful fact that we will repeatedly use is that the letter distribution at any other position in S' is independent of the letter distribution at position x. To formalize this, we introduce the function f that maps each position $i \in S$ to its unique corresponding position in S' if one exists. This is done by referencing the homologous path: if x is not deleted, then its corresponding position turns out to be the point (x,y) on the path with the largest y-value. The definition below formalizes this.

Definition A.2 Define the function $f : \{1,\ldots,|S|\} \longrightarrow \{1,\ldots,|S'|\} \cup \{\emptyset\}$ such that

$$f(x) = \begin{cases} \emptyset, & \text{if } x \notin [p+1, p+m'] \text{ or } x \text{ is deleted,} \\ \max\{y : (x,y) \in P_H\}, & \text{otherwise.} \end{cases}$$

If in the mutation process, position $x \in [p+1, p+, m']$ of S is not deleted, then either no mutation occurred at x or another character is substituted for $S[x]$. In either case, there exists a position y in S' such that $(x,y) \in P_H$, meaning that $\max\{y : (x,y) \in P_H\}$ is well-defined.

Below, we provide a simple lemma stating that if f takes the same value on two inputs, then it must map those inputs to $\emptyset$. In other words, f is injective on preserved positions of S.

Lemma A.1 For positions $x,y \in [|S|]$, either $f(x) = f(y) = \emptyset$ or $f(x) \neq f(y)$.

Proof If x and y are both deleted, then $f(x) = f(y) = \emptyset$. Otherwise, if only one of x or y is deleted, without loss of generality, let it be x, then $f(x) = \emptyset \neq f(y)$. Lastly, if neither x nor y is deleted, then they have unique corresponding positions x', y' on S', implying that $f(x) = x' \neq y' = f(y)$. □

As an example, consider again $S = TACTTCGC$, which is transformed into $S' = TACTTTAC$ in Fig. 1C. Consider position 4 on S: to calculate $f(4)$, we find the point $(4,y)$ on P_H (refer to Fig. A.1) with the largest y-value. This is the point $(4,5)$, so $f(4) = 5$. Since the letter T is inserted to the left of position 4 and position 4 is not deleted, the corresponding position on S' is 5. This confirms that $f(4)$ is the position on S' that corresponds to the position 4 on S. Now consider position 5 on S: this letter is deleted when generating S' so it has no corresponding position. So, $f(5) = \emptyset$, matching our intuition.

With f defined, we are equipped to analyse the dependency graph between S and S'. We derive three key results: sufficient conditions for a set of match variables, defined in the next section, to be independent of each other, sufficient conditions for two anchors to be independent of each other, and we bound the probabilities of the three anchor types listed in Sec. A.

Dependence structure between S and S'

The general strategy of our proof is to show that with high probability there are no spurious anchors and that there are not large uncovered gaps in the homologous path before the first anchor or after the last anchor in any optimal chain. To do this, we will need tail bounds for the number of spurious anchors N_S. In turn, this requires bounding the variance of N_S. To show that large uncovered gaps in the homologous path do not exist, we need to show that homologous anchors are sufficiently dense. For this, we will use concentration inequalities, which require bounding the expected value of the number of homologous anchors N_H. To these ends, we must understand the dependence structure between S and S', which is the purpose of this section.

We present three main results in this section: (1) we introduce the match graph, which captures the dependence structure between S and S' and prove a sufficient condition for a set of match variables, which are defined shortly, to be independent, (2) we use this independence

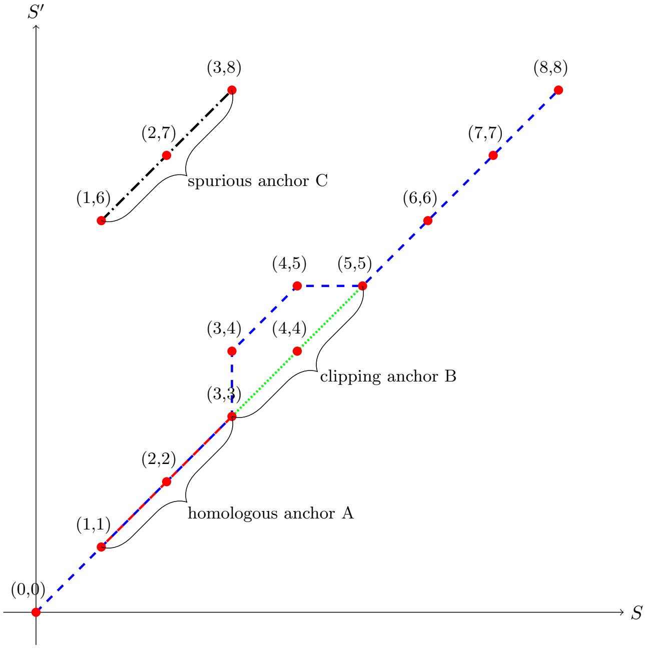

Fig. A.1. The points along the dashed blue line make up the homologous path given the edits turning $S = TACTTCGC$ into $S' = TACTTTAC$ following Fig. 1C. In this example, anchors are matching seeds of length 3. Anchor A (red dash) is a *homologous* anchor since it lies entirely on the path. Anchor B (green dash) is a *clipping* anchor since it lies partially on the path, namely, the midpoint of the anchor does not belong to the homologous path. Anchor C (black dash) is spurious since it lies entirely off the path.

result to bound the probabilities of each anchor type occurring, and (3) we prove a sufficient condition for two anchors to be independent.

The match graph, defined below, is a bipartite graph that captures the dependence structure between positions on S and S'. The original (also called the unconditioned) match graph contains edges between corresponding positions i in S and $f(i)$ in S' provided that $f(i) \neq \emptyset$. The intuition is that positions connected by an edge have dependent letter distributions; this is clear in the unconditioned match graph, since $\Pr(S'[f(i)] = S[i]) = 1 - \theta_s \neq \frac{1}{\sigma}$. We refer to *match variables*, where a match variable $M(i,j)$ is the event that $S[i] = S'[j]$. Fig. A.2 shows the case where S' is obtained through substitutions from S and the graph is conditioned on $A(1,3), A(3,1), A(2,4), A(4,2)$: note that $S[1] \to S'[3] \to S[3] \to S'[1] \to S[1]$ is a cycle in this induced match graph.

Definition A.3 Let $\mathcal{M} = \{M(a_1,b_1),\dots,M(a_p,b_p)\}$ be a set of matching variables where a_i are positions in S and b_i are positions in S'. The match graph induced by $\mathcal{M}$ refers to the graph $G = (V,E)$ where the vertices V are the letters in S and S', and the edges are given by $E = \{(x_i, y_{f(x_i)}) \mid i \in (p+1, p+m') \wedge f(x_i) \neq \emptyset\} \cup \{(x_h, y_l) \mid M(h,l) \in \mathcal{M}\}$. Note that $G(\mathcal{M})$ is bipartite.

Intuitively, if the match variables form a cycle, then they are dependent: in Fig. A.2, if $A(1,1) = A(1,3) = A(3,3) = 1$, then we must also have $A(3,1) = 1$. The first main result of this section is a converse of this statement: if the match variables do not induce a cycle in the match graph then they are independent.

We will use this fact to calculate the probability that different anchor types occur, and when a pair of anchor indicator variables are independent.

We begin with a lemma from Yu and Shaw (Shaw and Yu 2023), where they prove that if random variables belong to different connected components of the match graph then they are conditionally independent.

Lemma A.2 ((Yu and Shaw) Supplemental Lemma: Conditional independence in the match graph) Consider a set of random variables of the form $\mathcal{M} = \{M(i_1,j_1),\dots M(i_\ell,j_\ell)\}$. If two vertices u,v lie in separate connected components of $G(\mathcal{M})$, then they are conditionally independent of $\mathcal{M}$.

Proof Refer to Supplemental Lemma S2 of the prequel (Shaw and Yu 2023) for the proof of this result. □

Below, we prove two lemmas that will be combined to show that match variables are independent whenever the induced match graph does not contain a cycle. The first lemma, appearing below, establishes that a set of non-corresponding variables M_C, those $M(i,j)$ for which $f(i) \neq j$, are conditionally independent of any set of corresponding variables given that M_C does not induce a cycle in the conditional match graph. We will use this as one of the cases in

Lemma A.3 (Adapted from Yu and Shaw) Given a set of non-corresponding match variables $\mathcal{M} = \{M(i_1,j_1), M(i_2,j_2),\dots\}$, such that $f(i_\ell) \neq j_\ell$ for all $M(i_\ell,j_\ell) \in \mathcal{M}$ and a set of corresponding match variables $\mathcal{M}_C = \{M(a_1,b_1), M(a_2,b_2),\dots\}$, such that $f(a_t) = b_t$ for all

$M(a_t,b_t)\in\mathcal{M}_C$, then the match variables in $\mathcal{M}$ are independent conditioned on the match variables in $\mathcal{M}_C$ if the induced match graph has no cycles. Specifically, $\Pr(\mathcal{M}\mid\mathcal{M}_C)=\prod_{\ell}^{|\mathcal{M}|}\Pr(M(i_\ell,j_\ell)\mid\mathcal{M}_C)=\prod_{\ell}^{|\mathcal{M}|}\Pr(M(i_\ell,j_\ell))$.

Proof As in the prequel, denote $M(i_\ell,j_\ell)=M_\ell$ and $\mathcal{M}^-=\{M_1,\ldots,M_n\}$ for brevity. We proceed by induction. When $|\mathcal{M}|=0$, the statement holds trivially. Assume the statement holds up to all $n\in\mathbb{N}$. If $|\mathcal{M}|=n+1$, we can write $\Pr(M_1,\ldots,M_{n+1}\mid\mathcal{M}_C)=\Pr(\mathcal{M}^-\mid\mathcal{M}_C)\Pr(M_{n+1}\mid\mathcal{M}_C,\mathcal{M}^-)$. By the inductive hypothesis, $\Pr(M_1,\ldots,M_n\mid\mathcal{M}_C)=\prod_{i=1}^{n}\Pr(M_i)$. It remains to show that $\Pr(M_{n+1}\mid\mathcal{M}_C,\mathcal{M}^-)=\Pr(M_{n+1})$.

We have that $\Pr(M_{n+1}\mid\mathcal{M}_C,\mathcal{M}^-)=\sum_{\alpha\in\Sigma}\Pr(S[i_{n+1}]=\alpha,S'[j_{n+1}]=\alpha\mid\mathcal{M}_C,\mathcal{M}^-)$.

Since the match variables do not induce a cycle, we must hve that $i_{n+1}\in S$ and $j_{n+1}\in S'$ belong to separate connected components in the conditional match graph. Thus, the previous sum equals $\sum_{\alpha\in\Sigma}\Pr(S[i_{n+1}]=\alpha\mid\mathcal{M}_C,\mathcal{M}^-)\Pr(S'[j_{n+1}]=\alpha\mid\mathcal{M}_C,\mathcal{M}^-)$.

We will show that $\Pr(S[i_{n+1}]=\alpha\mid\mathcal{M}_C,\mathcal{M}^-)=\Pr(S'[j_{n+1}]=\alpha\mid\mathcal{M}_C,\mathcal{M}^-)=\frac{1}{\sigma}$. As before, consider the cyclic permutation of letters C_σ on the alphabet Σ. For DNA this could be the map $A\to G\to C\to T$. Note that applying C_σ to all positions of S and S' preserves the value of all match variables. As such, C_σ is a probability preserving transformation. Thus, if we let A be the set of all string pairs (S,S') such that $S[i_{n+1}]=\alpha$ then $\Pr(A\mid\mathcal{M}_C,\mathcal{M}^-)=\Pr(C_\sigma(A)\mid\mathcal{M}_C,\mathcal{M}^-)=\cdots=\Pr(C_\sigma^{|\Sigma|-1}(A)\mid\mathcal{M}_C,\mathcal{M}^-)$. The sets $A,C_\sigma(A),\ldots,C_\sigma^{|\Sigma|-1}(A)$ partition the set of string pairs, we have that $\Pr(S[i_{n+1}]=\alpha\mid\mathcal{M}_C,\mathcal{M}^-)=\Pr(A\mid\mathcal{M}_C,\mathcal{M}^-)=\frac{1}{\sigma}$. By the same argument considering pairs where $S'[j_{n+1}]=\alpha$, we have that $\Pr(S'[j_{n+1}]=\alpha\mid\mathcal{M}_C,\mathcal{M}^-)=\frac{1}{\sigma}$.

Thus, $\Pr(M_{n+1}\mid\mathcal{M}_C,\mathcal{M}^-)=\frac{1}{\sigma}=\Pr(M_{n+1})$, which completes the proof. □

Now, we show that when the match variables $\mathcal{M}$ contain only those $M(i,j)$ for which $f(i)=j$, i.e. the position j in S' corresponds to the position i in S during the generation process, then the random variables in $\mathcal{M}$ are independent.

Lemma A.4 The random variables

$$\mathcal{M}=\{M(i_1,j_1),M(i_2,j_2),\ldots\},$$

where $f(i_\ell)=j_\ell$ for all $M(i_\ell,j_\ell)\in\mathcal{M}$ are independent.

Proof Note that conditioning on these match variables does not add new edges to the unconditioned match graph.

Each position $i\in S$ has at most one corresponding position in S' and, similarly, each position $j\in S'$ has at most corresponding position in S. Thus, each $M(i_\ell,f(i_\ell))$ belongs to its own connected component. By Lemma A.2, the match variables are independent, i.e. $\Pr(\mathcal{M})=\prod_{\ell=1}^{|\mathcal{M}|}\Pr(M(i_\ell,j_\ell))$. □

Combining the two previous lemmas, we conclude that match variables $\mathcal{M}$ are independent if they do not induce a cycle in the match graph. This is detailed below.

Lemma A.5 The random variables

$$\mathcal{M}=\{M(i_1,j_1),M(i_2,j_2),\ldots\},$$

are independent if the induced match graph has no cycles.

Proof We first show the result holds for simple cases:

(1) If $f(i_\ell)\neq j_\ell$ for all $M(i_\ell,j_\ell)\in\mathcal{M}$, then this lemma reduces to showing that match variables with spurious positions that do not form a cycle are independent. This is proven in Lemma A.3.

(2) If $f(i_\ell)=j_\ell$ for all $M(i_\ell,j_\ell)\in\mathcal{M}$, then we must show that match variables of corresponding positions that do not induce a cycle are independent. This is proven in Lemma A.4.

Assume now that $\mathcal{M}$ contains both corresponding and non-corresponding match variables. Denote the set of non-corresponding match variables in $\mathcal{M}$ by $\mathcal{A}=\{M(i_\ell,j_\ell)\in\mathcal{M}\mid f(i_\ell)\neq j_\ell\}$ and the set of corresponding match variables by $\mathcal{M}$ by $\mathcal{B}=\{M(i_\ell,j_\ell)\in\mathcal{M}\mid f(i_\ell)=j_\ell\}$. For simple indexing, let $I(\mathcal{B})=\{\ell\mid M(i_\ell,j_\ell)\in\mathcal{B}\}$ and $I(\mathcal{A})=\{\ell\mid M(i_\ell,j_\ell)\in\mathcal{A}\}$ be the two index sets.

We have $\Pr(A,B)=\Pr(A\mid B)\Pr(B)$. By Lemma A.4, $\Pr(B)=\prod_{t\in I(\mathcal{B})}\Pr(M(i_t,j_t))$. By Lemma A.3, $\Pr(A\mid B)=\prod_{\ell\in I(\mathcal{A})}\Pr(M(i_\ell,j_\ell))$.

Thus, $\Pr(\mathcal{M})=\prod_{\ell\in I(\mathcal{A})}\Pr(M(i_\ell,j_\ell))\prod_{t\in I(\mathcal{B})}\Pr(M(i_t,j_t))=\prod_{p=1}^{|\mathcal{M}|}\Pr(M(i_p,j_p))$, concluding the proof. □

We now move to the second main result of this section, which is that the match graph induced by a single anchor is acyclic. From this it follows that the match variables present in an anchor are independent. Using this, we will bound the probabilities of each anchor type.

Lemma A.6 The match graph induced by a single anchor, $A(i,j)$, has no cycles.

Proof First note that any position in S that is deleted and any position in S' that is inserted can have degree at most 1, coming from the anchor $A(i,j)$. Any such position cannot be involved in a cycle, so we can remove all such positions from the graph. Relabel the surviving positions in S and S' as their new indices. The interval $(i,i+k-1)$ becomes $X_{i'}$, possibly empty, and $(j,j+k-1)$ becomes $X_{j'}$, also possibly empty. If $X_{i'}$ is empty then there cannot be any cycle that uses positions on S since each node has degree at most 1, and hence there are no cycles since the graph is bipartite; we conclude the same if $X_{j'}$ is empty. Note that the unconditioned match graph at this point appears as two sets of vertices of equal size with edges between each corresponding pair.

We now assume $X_{i'}$ and $X_{j'}$ are nonempty and that there exists a cycle. Let $i'=\min H_{i'}$ and $j'=\min X_{j'}$. First, suppose that $i'<j'$.

Let x_{i+l} be the first point on S belonging to a cycle with $l \geq 0$. Then x_{i+l} has the neighbor y_{i+l}, so let the first edge of the cycle be (x_{i+l}, y_{i+l}). Then y_{i+l} must have degree exactly 2 for there to exist a cycle and its remaining edge must be induced from the anchor. Since $i' < j'$, its neighbor lies to the left of x_{i+l}, it is some x_{i+a} where $0 \leq a < l$, contradicting the minimality of l. Similarly, if $i' > j'$, we can apply the same argument but for the largest l such that x_{i+l} is in a cycle. Thus, no cycle exists. □

The above lemma allows us to conclude that the probability a spurious anchors is $\frac{1}{\sigma^k}$.

Corollary A.1 (Spurious anchor probability) Let $(i,j) \in |S| \times |S'|$: if (i,j) is such that $\{(i+l, j+l) \mid 0 \leq l \leq k-1\} \cap P_H = \emptyset$, then $\Pr(A(i,j)) = \frac{1}{\sigma^k}$.

Proof Lemma A.6 shows that the match graph conditioned on a single anchor does not contain a cycle, which means that for an anchor $\Pr(A(i,j)) = \prod_{t=0}^{k-1} \Pr(M(i+t, j+t))$.

If (i,j) represents the start of a spurious anchor, then $\Pr(M(i+t, j+t)) = \frac{1}{\sigma}$ for each $0 \leq t \leq k-1$. Combining terms proves the lemma. □

We now give sufficient conditions for two anchors $A(i,j), A(h,l)$ to be independent; that is, $\Pr(A(i,j), A(h,l)) = \Pr(A(i,j))\Pr(A(h,l))$. Intuitively, the first condition $|i-h| \geq k$ or $|j-l| \geq k$ ensures that the anchors do not overlap too much – the anchors' coverage can overlap on S or S' but not on both. The second condition prevents 'twisting': consider anchors $A(1,3)$ and $A(3,1)$ in the substitution-only mutation model as shown in Fig. A.2. There exists a cycle going from $x_1 \to y_3 \to x_3 \to y_1 \to x_1$ where x_i represents node i on the top set of vertices and y_i represents node i on the bottom set of vertices. The biological interpretation is that under low mutation rates, x_1 and y_1 are likely to be equal, as are x_3 and y_3. This implies that the events $x_1 = y_3$ and $x_1 = y_3$ are not independent.

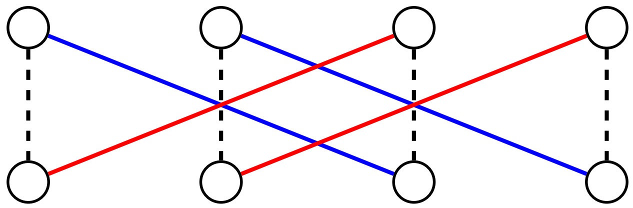

Fig. A.2. Induced match graph in the substitution-only regime of an initial string of length 4 with anchors $A(1,3)$ and $A(3,1)$. These anchors violate Yu and Shaw's (Shaw and Yu 2023) conditions for independence and, as can be seen, there exists a cycle in the graph.

Lemma A.7 (General independence lemma) For $A(i,j)$ and $A(h,l)$, if both of the following conditions hold:

1. $|i-h| \geq k$ or $|j-l| \geq k$, and
2. $[i : i+k-1] \cap f^{-1}([l : l+k-1]) = \emptyset$ or $[h : h+k-1] \cap f^{-1}([j : j+k-1]) = \emptyset$,

then the induced match graph on the M variables for $A(i,j)$ and $A(h,l)$ has no cycles.

Proof There are four cases to consider. We proceed with the first case, $|i-h| \geq k$ and $f[i : i+k-1] \cap [l : l+k-1] = \emptyset$. The remaining cases follow symmetrically.

Let $X_i = [i : i+k-1]$. Since $|i-h| \geq k$, we have that $[i : i+k-1] \cap [j : j+k-1] = \emptyset$, which implies that $f([i : i+k-1]) \cap f([h : h+k-1]) = \emptyset$ by Lemma A.1. Furthermore, since $f([i : i+k-1]) \cap [l : l+k-1] = \emptyset$, we have that for any $x \in X_i$, $f(x) \notin f([h : h+k-1]) \cup [l : l+k-1]$. Thus, $f(x)$ can have degree at most 2: one edge from the unconditioned match graph and one due to conditioning on $A(i,j)$. Similarly, all edges touching x must come from the original unconditioned match graph or from conditioning on one of the anchors. Since $|i-h| \geq k$, x has no edge due to conditioning on $A(h,\ell)$, and so x has degree at most 2. With this in place, we continue with the proof.

Assume that there exists a cycle C. Let $C(X_i) \subset X_i$ be the set of all points in X_i in the cycle. If $C(X_i) = \emptyset$, then there still exists a cycle after removing all edges induced by $A(i,j)$. This implies that there is a cycle in the match graph after conditioning on $A(h,\ell)$, contradicting our previous lemma. Thus, $C(X_i)$ is not empty.

We first show that for any $x \in C(X_i)$, x cannot be deleted. This is clear since if x were deleted then it has no edge in the unconditioned match graph and so can only have degree 1 due to some edge coming from conditioning on $A(i,j)$; since a vertex with degree 1 cannot be in a cycle, it follows $x \notin C(X_i)$. This is also establishes that $f(x)$ is not null.

Now, note that each $x_p \in C(X_i)$ has exactly two neighbors in the cycle: $f(x)$ and y_{j+p-i}, the neighbor from conditioning on $A(i,j)$. Specifically, there are $|C(X_i)|$ neighbors due to $A(i,j)$ in $C(X_i)$, each of which must be in the cycle. Each position in $f(C(X_i))$ has a neighbor in the cycle due to $A(i,j)$ and there are exactly $|f(C(X_i))| = |C(X_i)|$ such neighbors. Since each neighbor of $f(C(X_i))$ due to $A(i,j)$ is in X_i and in the cycle, this is exactly $C(X_i)$. This also implies that all neighbors of $C(X_i)$ due to edges from $A(i,j)$ are exactly the points in $f(C(X_i))$.

Considering the subgraph given by $C(X_i) \cup f(C(X_i))$. All vertices in this graph have degree exactly 2. Since all 2-regular graphs contain a cycle, it follows that this subgraph contains a cycle. However, it is a subgraph of the original match graph conditioned on $A(i,j)$, implying that it too contains a cycle, which cannot be true. This completes the proof. □

In Sec. 2.7, we bound the number of unrecovered points before the first anchor or after the last anchor in any optimal chain. This requires showing that homologous anchors are dense in the homologous path P_H. The key lemma in that section makes use of k-independence of special homologous anchors: those resulting from k positions in which no mutation occurs. The last portion of this section proves this fact; namely, that the indicator variables representing no mutation occurring in k consecutive positions are independent as long as they do not overlap in S.

Lemma A.8 Let E_i be the event that there are no mutations at position i for $i \in [p+1, p+m']$. If M is a collection of these E_i such that no two overlap in S, then the variables in M are independent.

Formally, for any collection $M = \{E_i\}_{i\in S}$ where $S \subset [p+1, p+m']$, such that $\forall i,j \in S$ if $i \neq j$ then $|i-j| \geq k$, then $\Pr(\cap_{i\in S} E_i) = \prod_{i\in S} \Pr(E_i)$.

Proof Since mutations occur independently at each position, $\Pr(\cap_{i\in S} E_i) = \prod_{i\in S} \Pr(E_i) = ((1-\theta_i)(1-\theta_d)(1-\theta_s))^{|S|} \geq (1-\theta_T)^{|S|}$. □

The following corollary is an immediate application of the previous lemma: a k-mer in the generative region of S has no mutations with probability $((1-\theta_i)(1-\theta_d)(1-\theta_s))^k$.

Corollary A.2 (No mutation in k-mer probability) Let $E_{i:i+k-1}$ be the event that there are no mutations in $S[i:i+k-1]$ for $i \in [p+1, p+m']$. Then $\Pr(E_{i:i+k-1}) = ((1-\theta_i)(1-\theta_d)(1-\theta_s))^k$

Proof This follows directly from the previous lemma: $\Pr(E_{i:i+k-1}) = \prod_{\ell=i}^{i+k-1} \Pr(E_\ell) = ((1-\theta_i)(1-\theta_d)(1-\theta_s))^k$. □

Applying the previous lemma to non-overlapping k-mers in S yields a result that we sought to prove: the events that non-overlapping k-mer regions in S have no mutations are independent.

Corollary A.3 (Independence of non-overlapping k-mers) Let $E_{i:i+k-1}$ be the event that there are no mutations in $S[i:i+k-1]$ for $i \in [p+1, p+m']$. If M is a collection of these $E_{i:i+k-1}$ such that no two overlap in S, then the variables in M are independent.

Proof Note that $\Pr(E_{i:i+k-1}) = \Pr(E_i, \ldots, E_{i+k-1})$. If $E_{i:i+k-1}$ and $E_{j:j+k-1}$ do not share any E_ℓ, they are independent.

For any $i,j \in S$ such that $i \neq j$, then $|i-j| \geq k$. This implies that $E_{i:i+k-1}$ and $E_{j:j+k-1}$ do not overlap: they contain distinct random variables. Applying the previous lemma gives the result. □

B. Anchor-count technical lemmas

Lemma B.1 For $t_0 = \frac{1}{2}\ln(\frac{9}{1+8\gamma})$, $e^{t_0}\rho'_i < 1$ and $(1-\theta_i) + \theta_i(\frac{(1-\rho'_i)e^t}{1-e^t\rho'_i}) \leq e$ for all $0 < \rho'_i < \gamma$.

Proof From the choice of t_0, it follows that $e^{t_0} \leq \frac{9}{1+8\gamma}$. Note that $f(x) = \frac{x}{1+\gamma(x-1)}$ is increasing for $x > 0$. We have $9 < \frac{e-1}{0.206} + 1 \leq \frac{e-1}{\theta_i} + 1$ because $\theta_i \leq \theta_T < 0.206$. Letting $A = \frac{e-1}{\theta_i} + 1$, the previous two facts give that $e^{t_0} \leq \frac{A}{1+\gamma(A-1)}$, and since $\rho'_i \leq \gamma$, we have additionally that $e^{t_0} \leq \frac{A}{1+\gamma(A-1)} \leq \frac{A}{1+\rho'_i(A-1)}$.

Multiplying both sides of this inequality by ρ'_i gives $e^{t_0}\rho'_i \leq \frac{A\rho'_i}{\rho'_i A + (1-\rho'_i)} < 1$ since $\rho'_i < \gamma < 1$. This final inequality shows why it is necessary to bound ρ'_i away from 1.

The second inequality in the lemma follows by rearrangement and inserting the expression represented by A:

Expanding the inequality gives $(1-\rho'_i)e^{t_0} + e^{t_0}\rho'_i A \leq A$, rearranging we get $(1-\rho'_i)e^{t_0} \leq A(1-\rho'_i e^{t_0})$. Simplifying yields $\frac{(1-\rho'_i)e^{t_0}}{1-\rho'_i e^{t_0}} \leq A$. Plugging in $A = \frac{e-1}{\theta_i} + 1$ and multiplying both sides by θ_i gives $\theta_i(\frac{(1-\rho'_i)e^{t_0}}{1-\rho'_i e^{t_0}}) \leq e - 1 + \theta_i$, from which we immediately get $(1-\theta_i) + \theta_i(\frac{(1-\rho'_i)e^t}{1-e^t\rho'_i}) \leq e$. This proves the second claim. □

Lemma B.2 (**Bounded Expansion (E)**) With probability $\geq 1 - 1/n$, no k-mer in $S[p+1:p+m']$ has more than $\frac{1}{t_0}(\frac{2}{\beta}+1)k$ inserted base pairs, for $t_0 = \frac{1}{2}\ln(\frac{9}{1+8\gamma})$.

Proof Denote the random variable representing the total insertion length at the $p+j$-th coordinate as I_j where

$$I_j = \begin{cases} 0, & \text{with probability } 1-\theta_i, \\ \text{Geom}(1-\rho'_i), & \text{with probability } \theta_i, \end{cases}$$

In particular, for $\ell > 0$, $\Pr(I_j = \ell) = \theta_i(1-\rho'_i)(\rho'_i)^{\ell-1}$. Define $Z = \sum_{j=1}^k I_j$, which represents the total insertion length, the expansion, of the first k-mer. A simple Chernoff bound shows that for any $t > 0$,

$$\Pr(Z \geq c) \leq \frac{\mathbb{E}[e^{tZ}]}{e^{tc}} = \frac{\prod_{j=1}^k \mathbb{E}[e^{tI_j}]}{e^{tc}} = \frac{\mathbb{E}[e^{tI_1}]^k}{e^{tc}}.$$

Where the first equality follows since the $\{I_j\}_{j=1}^k$ are independent and the second from them being identically distributed. Choosing

$t = t_0 = \frac{1}{2}\ln(\frac{9}{1+8\gamma})$, as in the previous lemma, we can calculate the moment generating function of I_1 directly:

$M_{I_1}(t_0) = \mathbb{E}[e^{t_0 I_1}] = (1-\theta_i) + \sum_{j=1}^{\infty}\theta_i(1-\rho_i')(\rho_i')^{j-1}e^{t_0 j} = (1-\theta_i) + \theta_i(1-\rho_i')e^{t_0}\sum_{j'=0}^{\infty}(\rho_i' e^{t_0})^{j'}$. The last term is a geometric series which converges since $\rho_i' e^t < 1$ by Lemma B.1. Thus, $M_{I_1}(t) = 1-\theta_i+\theta_i\frac{(1-\rho_i')e^{t_0}}{1-\rho_i' e^{t_0}}$, which the previous lemma (Lemma B.1) shows is at most e.

Thus, $\Pr(Z \geq c) \leq e^{k-t_0 c}$ for any $c \in \mathbb{R}$. Choosing $c = \frac{1}{t_0}(\frac{2}{\beta}+1)k$ gives $\Pr(Z_1 \geq \frac{1}{t_0}(\frac{2}{\beta}+1)k) \leq e^{k-(\frac{2}{\beta}+1)k} = e^{-\frac{2}{\beta}k} = e^{-2C\ln(n)} \leq e^{-2\ln(n)} = \frac{1}{n^2}$ since $C > 1$.

Define Z_i to be the random variable denoting the expansion of the $S[p+i:p+i+k-1]$, the i-th k-mer in $S[p+1:p+m']$, formally, Z_i is the sum of the insertion lengths at each position in the k-mer $S[p+i:p+i+k-1]$. Note that each Z_i has the same distribution as Z.

A simple union bound shows that $\Pr(\exists j : Z_j \geq \frac{1}{t_0}(\frac{2}{\beta}+1)k) \leq (m'-k+1)\frac{1}{n^2} \leq n\frac{1}{n^2} = \frac{1}{n}$, and the result follows. □

Lemma B.3 (Bounded Contraction (C)) With probability $\geq 1-1/n$, no ℓ-mer in $S[p+1:p+m']$ contracts to size $\leq \frac{(1-\theta_d)\ell}{2}$, where $\ell = \frac{21k}{\beta}$.

Proof The proof follows similarly to the previous lemma. Define

$$X_j = \begin{cases} 0, & \text{if the } j\text{-th index of } S[p+1:p+m'] \text{ is deleted (with probability } \theta_d), \\ 1, & \text{otherwise.} \end{cases}$$

Note if $X_j = 1$, then the j-th index of $S[p+1:p+m']$ survives. Let $X = \sum_{j=1}^{\ell} X_j$ be the total number of surviving indices in the ℓ-mer and set $q_d = 1-\theta_d$ to be the survival rate of an index. A classic Chernoff bound on the sum of i.i.d. Bernoulli random variables gives for any $0 < \delta < 1$: $\Pr(X \leq (1-\delta)q_d\ell) \leq \exp(-\frac{\delta^2 q_d \ell}{2})$. Since $\ell = ck = c\beta C\ln(n)$ where $c = \frac{21}{\beta}$ gives $\frac{\delta^2}{2}q_d c_0 C\beta \geq \frac{1}{8}(.794)21 > 2$, when $\delta = 1/2$ and $\theta_d \leq \theta_T \leq 0.206$. Thus, $\Pr(X \leq \frac{1}{2}q_d\ell) \leq \exp(-2\ln(n)) = \frac{1}{n^2}$.

A union bound over all ℓ-mers in $S[p+1:p+m']$ gives:

$$\Pr(\exists \ell\text{-mer shrunk to less than } \frac{(1-\theta_d)\ell}{2}) \leq n/n^2 = \frac{1}{n}.$$

□

Proof of Lemma 2 Let $S_p = \{(i,j) \in S \times S' \mid [(i,j),\ldots,(i+k-1,j+k-1)] \cap P_H = \emptyset\}$ be the set of all positions where an anchor at that position does not intersect the homologous path.

Define $B_k(i,j) = \{(h,l) \in S_p \mid |h-i| \leq k \cap |l-j| \leq k\}$, and

$$P_k(i,j) = \{(h,l) \in S_p : f^{-1}([l:l+k-1]) \cap [i:i+k-1] \neq \emptyset,$$
$$f^{-1}([j:j+k-1]) \cap [h:h+k-1] \neq \emptyset\}.$$

By Lemma A.7, for any $(h,l) \notin B_k(i,j) \cup P_k(i,j)$, $A(i,j)$ and $A(h,l)$ are independent. Then, $N_S = \sum_{(i,j)\in S_p} A(i,j)$. We calculate the variance as follows:

$$\begin{aligned} N_S^2 &= \sum_{(h,l)\in S_p}\sum_{(i,j)\in S_p} A(i,j)A(h,l) \\ &= \underbrace{\sum_{(i,j)\in S_p}\sum_{(h,l)\in S_p\setminus\left(B_k(i,j)\cup P_k(i,j)\right)} A(i,j)A(h,l)}_{S_1} + \underbrace{\sum_{(i,j)\in S_p}\sum_{(h,l)\in S_p\cap B_k(i,j)} A(i,j)A(h,l)}_{S_2} \\ &\quad + \underbrace{\sum_{(i,j)\in S_p}\sum_{(h,l)\in\left(S_p\cap P_k(i,j)\right)\setminus B_k(i,j)} A(i,j)A(h,l)}_{S_3}. \end{aligned}$$

Dealing first with S_1: by the independence lemma (Lemma A.7), $A(h,l), A(i,j)$ are independent, so:

$$\begin{aligned} \mathbb{E}(S_1) &= \sum_{(i,j)\in S_p}\sum_{(h,l)\in S_p\setminus(B_k(i,j)\cup P_k(i,j))} \mathbb{E}(A(i,j))\mathbb{E}(A(h,l)) \\ &\leq \sum_{(h,l)\in S_p}\sum_{(i,j)\in S_p} \mathbb{E}(A(i,j))\mathbb{E}(A(h,l)) = \mathbb{E}(N_S)^2. \end{aligned}$$

Note $\mathbb{E}(A(h,l)A(i,j)) \leq \mathbb{E}(A(i,j))$ since the anchor random variables take values in $\{0,1\}$. Using that $|B_k(i,j)| \leq 4k^2$, $\Pr(A(i,j)=1) = \frac{1}{\sigma^k}$ for $(i,j) \in S_p$ from Corollary A.1, and the naive bound $|S_p| \leq mn$, we get:

$$\mathbb{E}(S_2) \leq \sum_{(i,j)\in S_p}\sum_{(h,l)\in S_p\cap B_k(i,j)} \mathbb{E}(A(i,j)) \leq 4k^2\frac{mn}{\sigma^k}.$$

Lastly, we handle the S_3 term. Working under EC, the region $S[i:i+k-1]$ can expand to have at most $(\frac{1}{t_0}(\frac{2}{\beta}+1)+1)k+2k$

corresponding positions l on S'. Note the additional $2k$ comes from k-mers that start before and after the corresponding region but still intersect it. The same argument shows that there are at most $(\frac{1}{t_0}(\frac{2}{\beta}+1)+1)k+2k$ positions j that correspond to $S[h:h+k-1]$. Thus, $|P_k(i,j)| \le ((\frac{2}{\beta}+1)+1)k+2k)^2 = (\frac{1}{t_0}(\frac{2}{\beta}+1)+3)^2k^2 \le \frac{1}{2}T_0k^2$.

This yields,

$$\mathbb{E}(N_S^2) \le \mathbb{E}(N_S)^2 + T_0k^2\frac{mn}{\sigma^k}.$$

From which it immediately follows that $\text{var}(N_S) \le T_0k^2\frac{mn}{\sigma^k}$. □

Proof of Lemma 3 Call the event $X = \{N_S \ge n^{2-C} + \sqrt{T_0}C\log(n)n^{\frac{3-C}{2}}\}$, i.e. the event that there are more spurious anchors than the amount given by the expression. We are looking to bound $\Pr(X \mid EC)$

To do this, let us first show that $\frac{mn}{\sigma^k} \le n^{2-C}$. Recall that $k = C\log(n)$. Under EC, $m \le cm'$ for the expansion constant $c>0$. Since $m' \ll n$, it follows that $m \ll n$. Thus, $\log(m) \le \log(n)$. From this inequality, we get immediately that $(C-1)\log(n)+\log(m) \le C\log(n) = k$. Subtracting $(C-1)\log(n)$ and adding $\log n$ to both sides yields $\log(mn)-k \le (2-C)\log(n)$. Finally, $\log(\frac{mn}{\sigma^k}) \le \log(n^{2-C})$, which gives the inequality.

Now, by Chebyshev, $\Pr(N_S \ge \mathbb{E}(N_S \mid EC)+\sqrt{n\text{var}(N_S \mid EC)} \mid EC) \le \frac{1}{n}$. We can upper bound $\Pr(X \mid EC)$ by using the variance bound of N_S given by Lemma 2 and the previous inequality. This yields: $\text{var}(N_S \mid EC) \le T_0k^2\frac{mn}{\sigma^k}$, so $\frac{1}{n} \ge \Pr(N_S \ge \mathbb{E}(N_S \mid EC)+\sqrt{n\text{var}(N_S \mid EC)} \mid EC) \ge \Pr(N_S \ge \mathbb{E}(N_S \mid EC)+\sqrt{T_0}C\log(n)n^{\frac{3-C}{2}} \mid EC)$. Finally, bounding $\mathbb{E}(N_S \mid EC) \le \frac{mn}{\sigma^k}$ and using our previously shown inequality, we obtain:

$$\Pr\Big(N_S \ge n^{2-C} + \sqrt{T_0}C\log(n)n^{\frac{3-C}{2}} \mid EC\Big) \le \frac{1}{n}.$$

□

C. Runtime theorem proof

The runtime of seed-chain-extend T_{SCE} is the runtime of chaining T_{Chain} and extension T_{Ext}. Since we are using linear gap costs, the runtime of chaining will be $O(N\log N)$. To show that $\mathbb{E}(T_{SCE}) = O(mn^{C\alpha}\log n)$ for any optimal chain, we will first show that $\mathbb{E}(T_{Chain}) = O(mn^{C\alpha}\log n)$.

Lemma C.1 The expected runtime of chaining $\mathbb{E}(T_{Chain})$ for any optimal chain is $O(mn^{C\alpha}\log n)$.

Proof Under G, all anchors are homologous or clipping. We will obtain a loose bound on the total number of anchors under G. Note first that there can be at most m homologous anchors since $|S'| = m$. Each point on $|P_H|$ can be in at most k clipping anchors: it can be in any one of the k positions in a clipping anchor. Thus, there can be at most $k|P_H|$ clipping anchors. Under G, $|P_H| \le c|S'| = cm$ for some constant $c>0$. Thus, $N \le m+ckm$.

It follows that $N\log N \le (m+ckm)\log(m+ckm)$, and so $\mathbb{E}(N\log N \mid G) \le (m+ckm)\log(m+ckm)$. Since $\log(m+ckm) \ll \log(n)$ and $ck \ll n^{C\alpha}$, it follows that $\mathbb{E}(N\log N \mid G) = O(mn^{C\alpha}\log n)$.

We use a worst-case bound for the case where G^c occurs. Note that $N \le mn$ since there can be at most one anchor for each pair of positions in S,S'. Thus, $\mathbb{E}(N\log N \mid G^c)\Pr(G^c) \le mn\log(mn)\frac{6}{n} = O(m\log(mn)) = O(m\log(n))$.

By the law of total expectation, $\mathbb{E}(N\log N) = \mathbb{E}(N\log N \mid G)\Pr(G) + \mathbb{E}(N\log N \mid G^c)\Pr(G^c) \le O(mn^{C\alpha}\log n) + O(m\log(n)) = O(mn^{C\alpha}\log n)$.
□

The strategy for showing that $\mathbb{E}(T_{Ext}) = O(mn^{C\alpha}\log n)$ for an optimal chain will be to upper bound its extension runtime by that of the subchain of homologous anchors without any mutations occurring at 'nice' positions: $\{p+1, p+1+k, p+1+2k, \ldots\}$. Intuitively, this works because that subchain is sparser and extension through sparser subchains takes longer. We begin by showing that every mutation-free homologous anchor that lies between the first and last anchor in an optimal chain must belong to it.

Lemma C.2 (Working in ***G***) Let $\mathcal{C} = ((i_1,j_1),\ldots,(i_u,j_u))$ be an optimal chain. Under G, it consists only of homologous and clipping anchors. Then if there is an index $a \in [p+1, p+m'-k+1]$ such that $i_1 \le a \le i_u$ and there are no mutations in $\{a,\ldots,a+k-1\}$, then $(a,f(a)) \in C$.

Proof If $(a,f(a)) \in \mathcal{C}$, then the result is trivially true. Assume that $(a,f(a)) \notin \mathcal{C}$. We must show that adding it to $\mathcal{C}$ is both valid and increases the score of $\mathcal{C}$.

Turning to the first part: if adding $(a,f(a))$ breaks the anchor monotonicity property of the chain, then there exists some anchor (i,j) such that (i,j) and $(a,f(a))$ intersect the homologous path and either $i \le a$ and $j \ge f(a)$ or $i \ge a$ and $j \le f(a)$. We consider the first case, $i \le a$ and $j \ge b$, noting that the proof for the second case is identical. Since $(a,f(a))$ is homologous, all of its points lie on P_H. Furthermore, since there no mutations occur in the k positions $[a:a+k-1]$ on S, it follows that the points $(a+t, f(a)+t) \in P_H$ for $0 \le t \le k$. Since P_H touches the anchor (i,j), this means that there is a portion of P_H that connects $(a+k, f(a)+k)$ to some point $(x,y) \in \{(i+t, j+t) \mid 0 \le t \le k-1\}$. However, since $a \ge i$, it follows that $a+k > i+k-1$, a contradiction. It follows that $(a,f(a))$ can be added to $\mathcal{C}$.

Lastly, adding $(a,f(a))$ to $\mathcal{C}$ increases its score by 1, since there is no change in the linear-gap cost, but there is an additional anchor. □

The lemma above applies specifically to preserved homologous anchors; clipping anchors can be mutually exclusive. In particular, the homologous path can 'weave' between the two clipping anchors with the same x value, implying that both cannot be in the same chain.

The lemma below establishes that if two chains share a first and last anchor and one is a subchain of the other, then its extension runtime is greater.

Lemma C.3 (Extension takes longer for sparser chains) Let $\mathcal{C}=((i_1,j_1),...,(i_u,j_u))$ and $\mathcal{C}'=((i'_1,j'_1),...,(i'_u,j'_u))$ be two chains such that $(i_1,j_1)=(i'_1,j'_1)$ and $(i_u,j_u)=(i'_u,j'_u)$ and $\mathcal{C}'\subset\mathcal{C}$. Then the runtime of extension through $\mathcal{C}'$ is $\geq$ the runtime of extension through $\mathcal{C}$.

Proof The trivial cases are when $|\mathcal{C}|=|\mathcal{C}'|=1$ or $|\mathcal{C}|=|\mathcal{C}'|=2$, in which case they are the same chain and the result holds trivially. For the remainder of the proof, assume that $|\mathcal{C}|>2$.

Denote the runtime of extension in the subchain (i_a,j_a) to (i_b,j_b) of a chain $\mathcal{C}_0$ as $T_{Ext}((i_a,j_a):(i_b,j_b);\mathcal{C}_0)$. Then we can express the extension runtime of $\mathcal{C}$ as $T_{Ext}(\mathcal{C})=\sum_{\ell=1}^{u'-1}T_{Ext}((i'_\ell,j'_\ell):(i'_{\ell+1},j'_{\ell+1});\mathcal{C})$. In other words, the extension runtime of $\mathcal{C}$ can be decomposed as the sum of the extension runtimes of the subchains induced by $\mathcal{C}'$ since they share the same endpoints.

Since $T_{Ext}(\mathcal{C}')=\sum_{\ell=1}^{u'-1}T_{Ext}((i'_\ell,j'_\ell):(i'_{\ell+1},j'_{\ell+1});\mathcal{C}')$, it suffices to show that $T_{Ext}((i'_\ell,j'_\ell):(i'_{\ell+1},j'_{\ell+1});\mathcal{C})\leq T_{Ext}((i'_\ell,j'_\ell):(i'_{\ell+1},j'_{\ell+1});\mathcal{C}')$ for each $1\leq\ell\leq u'-1$.

Without loss of generality, let $\ell=1$. Denote the subchain of S between $(i'_1,j'_1),(i'_2,j'_2)$ as $((i_1,j_1),...(i_p,j_p))$ where $(i_p,j_p)=(i'_2,j'_2)$. Furthermore, let $G_\ell(S;\mathcal{C})=\max(i_{\ell+1}-i_\ell-k+1,0),G_\ell(S';\mathcal{C})=\max(j_{\ell+1}-j_\ell-k+1,0)$ be the gap size of the ℓ-th anchor on S and S'. The values $G_\ell(S;\mathcal{C}')$ and $G_\ell(S';\mathcal{C}')$ are defined similarly. Since $i'_2-i'_1=\sum_{\ell=1}^{p-1}i_{\ell+1}-i_\ell$, it is clear that if $i'_2-i'_1-k+1\leq 0$, then $G_\ell(S;\mathcal{C})=0$ for all $1\leq\ell\leq p-1$. Otherwise, it is also clear that $G_1(S;\mathcal{C}')=i'_2-i'_1-k+1\geq\sum_{\ell=1}^{p-1}G_\ell(S;\mathcal{C})$ and similarly, $G_1(S';\mathcal{C}')\geq G_1(S';\mathcal{C})$.

We have that $T_{Ext}((i_1,\ j_1)\ :\ (i_p,\ j_p);\ \mathcal{C})\ =\ \sum_{\ell=1}^{p-1}G_\ell(S;\ \mathcal{C})G_\ell(S';\ \mathcal{C})$. Since each gap is non-negative, this is $\leq$ $(\sum_{\ell=1}^{p-1}G_\ell(S;\mathcal{C}))(\sum_{\ell=1}^{p-1}G_\ell(S';\mathcal{C}))\ \leq\ G_1(S;\mathcal{C}')G_1(S';\mathcal{C}')$ which is exactly $T_{Ext}((i'_1,\ j'_1)\ :\ (i'_2,\ j'_2);\ \mathcal{C}')$. Thus, we have shown that $T_{Ext}((i_1,j_1):(i_p,j_p);\mathcal{C})\leq T_{Ext}((i'_1,j'_1):(i'_2,j'_2);\mathcal{C}')$, as desired.
□

Definition C.1 Recall that a preserved anchor is some anchor $(i,f(i))$ for which there are no mutations at any position in the k-mer $S[i:i+k-1]$. Define K to be the chain of all preserved anchors occurring at $(i,f(i))$ for $i\in\{p+1,p+1+k,...,p+1+(\lfloor\frac{S}{k}\rfloor-1)k\}$.

Define Y_i^K to be the random variable denoting the number of uncovered bases between a preserved anchor occurring at position $i\in\{p+1,p+1+k,...,p+1+(\lfloor\frac{S}{k}\rfloor-1)k\}$, if it exists, and the next preserved anchor in K. Otherwise, $Y_i^K=0$. Formally, $Y_i^K=\ell$ if positions $[i,...,i+k-1]$ and $[i+\ell+k,...,i+\ell+2k-1]$ on S are unmutated and there is no preserved anchor in between. If $(i,f(i))$ is not a preserved anchor, then $Y_i^K=0$.

With the tools developed in the previous two lemmas, we now prove that the extension through the convenient anchors K, with a particular start and end anchor chosen, takes longer than extending through $\mathcal{C}\supset K$.

Lemma C.4 (Working in $\boldsymbol{G}$) Let $\mathcal{C}$ be any optimal chain for S,S'. Define $G_{start}(S),G_{start}(S')$, to be the distance from the start of P_H to the first anchor of K in S and S', respectively, and if K is empty, then $G_{start}(S)=m',G_{start}(S')=m$. $G_{end}(S)$ and $G_{end}(S')$ are defined similarly.

Then $T_{ext}(\mathcal{C})\leq(G_{start}(S)+k)(G_{start}(S'))+T_{ext}(K)+(G_{end}(S)+k)(G_{end}(S'))$

Proof Let $\mathcal{C}=((i_1,j_1),...,(i_u,j_u))$. Since we are working in G, all of these anchors are homologous or clipping. It follows that $i_1\geq p-k+1$, since otherwise $\{(i_1+t,j_1+t)\,|\,0\leq t\leq k-1\}\cap P_H=\emptyset$ since $i_1+k-1\leq p-k+1+k-1=p<p+1$.

There are two cases to consider. The first is when K is empty, i.e. there are no anchors occurring at positions $p+1+kt$ in $S[p+1:p+m']$ for $0\leq t$. In this case, $G_{start}(S)=m',G_{start}(S')=m$, and the expression on the right hand side evaluates to $2(m+k)(m'+k)$. Since $i_1\geq p-k+1,j_1\geq 0$ and, similarly, $i_u\leq p+m'+k,j_u\leq m$, it follows that extending between (i_1,j_1) and (i_u,j_u) has runtime at most $(i_u-i_1)(j_u-j_1)\leq(m+2k)(m')\leq 2(m+k)(m')=2(G_{start}(S)+k)(G_{start}(S'))$ since $m,m'\gg k$ under G. This establishes the first case.

Assume now that K is non-empty. Let the first anchor in K be (i_p,j_p) and the last anchor be (i_q,j_q). If $|K|=1$, then they are the same. Define $T_{Ext}(K;\mathcal{C})$ to be the runtime of extension for anchors occurring between (i_p,j_p) and (i_q,j_q) in $\mathcal{C}$. This is well-defined, and $K\subset\mathcal{C}$ by Lemma C.2. Then by Lemma C.3, $T_{Ext}(K;\mathcal{C})\leq T_{Ext}(K)$. By Lemma C.3, we also have that the extension runtime of the chain $\mathcal{C}'=((i_1,j_1),(i_p,j_p))$ is at least that of the sub-chain containing all anchors from (i_1,j_1) up to and including (i_p,j_p); note that if $(i_1,j_1)=(i_p,j_p)$, then $\mathcal{C}'$ is technically not a valid chain but then the extension runtimes mentioned are both 0. Lastly, since $i_1\geq p+1-k$, it follows that the extension region defined by the endpoints $(p+1-k,0),(i_p,j_p)$ contains the region defined by endpoints $(i_1,j_1),(i_p,j_p)$, and so the extension runtime of the first box is at least $T_{Ext}(\mathcal{C}')$. The extension of the first region is exactly $(i_p-(p+1)+k)(j_p)=(G_{start}(S)+k)(G_{start}(S'))$. By the same argument applied to the extension after (i_q,j_q), we obtain the desired inequality. □

The only remaining task is to bound $\mathbb{E}(T_{Ext})$. This is done below by making use of the previous lemma and the law of total expectation with respect to the space G.

Lemma C.5 The expected runtime of extension $\mathbb{E}[T_{Ext}]$ for any optimal chain $\mathcal{C}$ is $O(mkn^{C\alpha})$.

Proof By linearity of expectation, $\mathbb{E}[T_{Ext}(\mathcal{C})]=\mathbb{E}[T_{Ext}(\mathcal{C})\mid G]\Pr(G)+\mathbb{E}[T_{Ext}(\mathcal{C})\mid G^c]\Pr(G^c)$. Using that $\Pr(G^c)\leq\frac{6}{n}$, $\mathbb{E}(T_{Ext}(\mathcal{C})\mid G^c)\Pr(G^c)\leq mn(\frac{6}{n})=O(m)$ since the runtime extension of $\mathcal{C}$ cannot exceed the runtime of a full naive alignment between S and S'. It suffices to prove then that $\mathbb{E}[T_{Ext}(\mathcal{C})\,|\,G]\Pr(G)=O(mkn^{C\alpha})$.

The general strategy to bound conditional expectations will be to first use the G space to rewrite the expectations, then to bound

the unconditional expectations that have convenient probability independence properties, and then to convert that to a bound on the conditional expectation.

In Lemma C.4, we showed that $\mathbb{E}(T_{Ext}(\mathcal{C})\,|\,G) \le \mathbb{E}[(G_{start}(S)+k)(G_{start}(S'))\,|\,G] + \mathbb{E}[T_{ext}(K)\,|\,G] + \mathbb{E}[(G_{end}(S)+k)(G_{end}(S'))\,|\,G]$. We show that each term is $O(mkn^{C\alpha})$, from which it follows that the sum is also $O(mkn^{C\alpha})$.

First, consider $\mathbb{E}[(G_{start}(S)+k)(G_{start}(S'))\mid G]$. Under G, $G_{start}(S') \le c(G_{start}+k)$ by Lemma 1, where c is the expansion constant; the number of points corresponding to the region in S before the first k-mer without a mutation cannot expand more than c times. The addition of k is to ensure that $G_{start}(S)+k \ge k$, which is necessary since Lemma 1 applies to k-mers. Thus, under G, $(G_{start}(S)+k)(G_{start}(S')) \le c(G_{start}(S)+k)^2$. We now work with the unconditional expectation $\mathbb{E}[c(G_{start}(S)+k)^2] = c(\mathbb{E}[G_{start}(S)^2] + 2k\mathbb{E}[G_{start}(S)] + k^2)$. Note that $G_{start} = \ell k$ for $\ell \ge 0$. We can check that $kG_{start}(S) \le G_{start}(S)^2$. If $G_{start}=0$, then both sides vanish, and otherwise $kG_{start}(S) = \ell k^2 \le (\ell k)^2 = G_{start}(S)^2$. Thus, $2k\mathbb{E}[G_{start}(S)] = O(\mathbb{E}[G_{start}(S)^2])$. We calculate this term now. Note that $\Pr(G_{start}(S)=\ell k) \le (1-((1-\theta_i)(1-\theta_d)(1-\theta_s))^k)^\ell((1-\theta_i)(1-\theta_d)(1-\theta_s))^k$. Writing $\theta_0 = (1-\theta_i)(1-\theta_d)(1-\theta_s) \ge 1-\theta_T$, is the probability that no mutation occurs at a position. With this simpler notation, $\Pr(G_{start}(S)=\ell k) \le (1-\theta_0^k)^\ell \theta_0^k$. It follows that

$$\mathbb{E}[G_{start}(S)^2] \le \theta_0^k \sum_{\ell=1}^{\infty} (\ell k)^2 (1-\theta_0^k)^\ell = O\Big(\frac{k^2\theta_0^k}{\theta_0^{3k}}\Big) = O(n^{2C\alpha}\log^2 n)$$

since $\theta_0 \ge 1-\theta_T$, and using the fact that for $|x|<1$, $\sum_{i=1}^{\infty} i^2 x^i = \frac{x(x+1)}{(1-x)^3}$. Since $m' = \Omega(n^{2C\alpha+\epsilon})$ and $k = O(\log n)$, it follows that $n^{2C\alpha}\log^2 n \ll m'$, so $\mathbb{E}[G_{start}(S)^2] = O(m')$. Since $k^2 = O(m')$, it follows that $\mathbb{E}[c(G_{start}(S)+k)^2] = O(m')$. By linearity of expectation, $\mathbb{E}[(G_{start}(S)+k)(G_{start}(S'))\mid G] \le \frac{\mathbb{E}[c(G_{start}(S)+k)^2]}{\Pr(G)} \le \frac{m'}{1-\frac{6}{n}} \le \frac{cm}{1-\frac{6}{n}}$, where we used that $m' \le cm$ under G by Lemma 1. Thus, $\mathbb{E}[(G_{start}(S)+k)(G_{start}(S'))\,|\,G] = O(m)$, and by the same argument, $\mathbb{E}[(G_{end}(S)+k)(G_{end}(S'))\,|\,G] = O(m)$.

We will now bound the term $\mathbb{E}[T_{ext}(K)\,|\,G]$. Under G, the corresponding distance in S' from position i to the start of the next k-mer without mutations is at most $c(Y_i^K+k)$ where c is the expansion factor. The addition of k comes from the fact that Y_i^K can be less than k and the EC lemma bounds the expansion of k-mer regions of S while generating S'. Thus, $\mathbb{E}[T_{ext}(K)\,|\,G] \le \sum_{i=p+1}^{p+m'} \mathbb{E}[cY_i^K(Y_i^K+k)\,|\,G] = \sum_{i=p+1}^{p+m'} c\mathbb{E}[(Y_i^K)^2\,|\,G] + \sum_{i=p+1}^{p+m'} k\mathbb{E}[Y_i^K\,|\,G]$. We will bound both of these terms separately and show that they are both $O(mkn^{C\alpha})$.

We first bound the unconditional version of the first term: $\sum_{i=p+1}^{p+m'} \mathbb{E}[(Y_i^K)^2]$. If $Y_i^K = \ell k$, then there are two regions of k consecutive positions without any mutations, and there are ℓk positions for which each k-mer starting at position $p+1+kt$ in S has some mutation. Thus, $\Pr(Y_i^K = \ell k) \le (1-\theta_i)(1-\theta_d)(1-\theta_s)^{2k}(1-((1-\theta_i)(1-\theta_d)(1-\theta_s))^k)^\ell$. Thus, $\Pr(Y_i^K = \ell k) \le \theta_0^{2k}(1-\theta_0^k)^\ell$. We get that $\sum_{i=p+1}^{p+m'} \mathbb{E}[(Y_i^K)^2] \le \frac{m'}{k}\sum_{\ell=1}^{\infty}(\ell k)^2\theta_0^{2k}(1-\theta_0^k)^\ell$ since there are at most $\frac{m'}{k}$ non-zero random variables Y_i^K for $i \in \{p+1, p+1+k, \ldots, \}$.

Again, using the fact that for $|x|<1$, $\sum_{i=1}^{\infty} i^2x^i = \frac{x(x+1)}{(1-x)^3}$, we get that $\frac{m'}{k}\sum_{\ell=1}^{\infty}(\ell k)^2\theta_0^{2k}(1-\theta_0^k)^\ell = \frac{m'}{k}\theta_0^{2k}k^2\frac{(1-\theta_0^k)(2-\theta_0^k)}{\theta_0^{3k}} \le \frac{m'k}{\theta_0^k}$. Using that $\theta_0 \ge 1-\theta_T$, we obtain $\sum_{i=p+1}^{p+m'}\mathbb{E}[(Y_i^K)^2] \le m'k(1-\theta_T)^{-k} = m'kn^{C\alpha}$.

Now, we will bound the second unconditional term: $\sum_{i=p+1}^{p+m'}\mathbb{E}[kY_i^K]$. If $Y_i^K = 0$, then $kY_i^K = (Y_i^K)^2 = 0$, and otherwise, $Y_i^K > k$. Thus, $kY_i^K \le (Y_i^K)^2$. It follows that $\sum_{i=p+1}^{p+m'}\mathbb{E}[kY_i^K] \le \sum_{i=p+1}^{p+m'}\mathbb{E}[(Y_i^K)^2] \le m'kn^{C\alpha}$.

Combining the two inequalities and using linearity of expectation yields that $\mathbb{E}(T_{Ext}\,|\,G) \le \sum_{i=p+1}^{p+m'}\mathbb{E}[cY_i^K(Y_i^K+k)\,|\,G] \le \frac{m'kn^{C\alpha}}{\Pr(G)} \le \frac{m'kn^{C\alpha}}{1-\frac{6}{n}} \le \frac{cmkn^{C\alpha}}{1-\frac{6}{n}} = O(mkn^{C\alpha})$ since $m' \le cm$ for the expansion constant c under G by Lemma 1.

Note that $mkn^{C\alpha} \gg m$, so we have shown that all three terms in the upper bound of $\mathbb{E}[T_{Ext}(\mathcal{C})\,|\,G]$ are $O(mkn^{C\alpha})$. We conclude then that $\mathbb{E}[T_{Ext}(\mathcal{C})\,|\,G] = O(mkn^{C\alpha})$, which completes the proof.
□

Having calculated $\mathbb{E}(T_{Chain})$ and $\mathbb{E}(T_{Ext})$ for an optimal chain, we immediately obtain the full runtime result below.

Theorem C.1 *The expected runtime $\mathbb{E}[T_{SCE}]$ of seed-chain-extend for any optimal chain C under the constraints given in Def. 3 is $O(mn^{C\alpha}\log n)$.*

Proof The runtime T_{SCE} of any chain $\mathcal{C}$ is $T_{Chain}+T_{Ext}$, the runtime of seeding the query S', chaining and the runtime of extension. Seeding S' is fast and takes $O(m)$ time.

Since $\mathcal{C}$ is an optimal chain, by Lemma C.1, $\mathbb{E}[T_{Chain}] = O(mn^{C\alpha}\log n)$, and by Lemma C.5, $\mathbb{E}[T_{Ext}] = O(mn^{C\alpha}\log n)$. Thus, $\mathbb{E}[T_{SCE}] = O(m) + O(\mathbb{E}[T_{Chain}]) + O(\mathbb{E}[T_{Ext}]) = O(mn^{C\alpha}\log n)$. □

D. Old recoverability and clipping anchors

The main difference between the analysis presented in this paper, where indels are present, and the prequel, which deals with the substitution-only model, is the presence of clipping anchors. The purpose of this section is to briefly describe some of the issues surfaced by clipping anchors, and the motivation of our recoverability definition to solve these issues. An overview of the issues will be given first, and proofs will follow.

Recall that a clipping anchor is an anchor that contains a point on the homologous path but does not entirely 'contain' the path between the start and end of the anchor. The primary difficulty from clipping anchors is that for large enough n, they are on the same order as the number of homologous anchors. In the setup shown in the paper, any optimal chain is all homologous and clipping anchors. Because of this, the expected number of points missed due to clipping anchors is on the order of $\mathbb{E}(N_C)$. However, $\mathbb{E}(N_C) = O(\frac{m}{k}n^{-C\alpha})$.

The initial recoverability definition we used was a simple extension of the one in the prequel: all points on the homologous path that lie in an anchor or an extension region are considered 'recovered', as shown below.

Definition D.1 (Initial definition of recoverability) Given a chain $\mathcal{C}=((i_1,j_1),...,(i_u,j_u))$, we define the union of all possible alignments for the chain $\mathcal{C}$, $Align(\mathcal{C})$, as:

$$Align(\mathcal{C})=\bigcup_{\ell=1}^{u}\{(i_\ell,j_\ell),...,(i_\ell+k-1,j_\ell+k-1)\}\cup\bigcup_{\ell=1}^{u-1}Ext(\ell).$$

Where $Ext(\ell)=\{i_\ell+k-1,...,i_{\ell+1}\}\times\{j_\ell+k-1,...,j_{\ell+1}\}$. If $i_\ell+k-1>i_{\ell+1}$ or $j_\ell+k-1>j_{\ell+1}$, then $Ext(\ell)=\emptyset$.

The recoverability of the chain, $R(\mathcal{C})$, is defined to be:

$$R(\mathcal{C})=\frac{|Align(\mathcal{C})\cap P_H|}{|P_H|}$$

Using this definition, we can bound the expected recoverability of any optimal chain as follows: $\mathbb{E}(R)=\mathbb{E}(R\,|\,G)\Pr(G)+\mathbb{E}(R\,|\,G^c)\Pr(G^c)\le\mathbb{E}(R\,|\,G)+\Pr(G^c)\le\mathbb{E}(R\,|\,G)+\frac{6}{n}$. In the G space, any optimal chain will contain all homologous anchors and, in the worst case, all clipping anchors. When this occurs, $\mathbb{E}(R\,|\,G)\le 1-\mathbb{E}(\frac{N_C}{k|P_H|})$. In other words, the number of missed points is at least the number of clipping anchors over k since there is at least one missed point on P_H for each clipping anchor and at most k clipping anchors can share any point. This is a vast undercount but suffices to show the difficulty of clipping anchors.

We will now lower bound $\mathbb{E}(\frac{N_C}{k|P_H|}\,|\,G)$. Under G, $|P_H|=cm$ for some $c>0$ due to bounded expansion and contraction (Lemma 1). Furthermore, $\mathbb{E}(N_C\,|\,G)\ge\frac{1}{\sigma}\theta_i\theta_d(1-\rho_i')\frac{m}{k}(1-\theta_T)^{k-1}=\frac{\theta_i\theta_d}{\sigma(1-\theta_T)}(1-\rho_i')\frac{m}{k}(1-\theta_T)^k$. We can rewrite this as $c'\frac{m}{k}(1-\theta_T)^k$ after absorbing constants into c'. This bound will also be shown rigorously, but the idea is that a clipping anchor can occur from having $k-1$ positions with no mutations and then the last position in the anchor has an insertion and deletion. Since there are many more ways for clipping anchors to occur, this is clearly a lower bound.

Returning to the recoverability bound, we have that $\mathbb{E}(\frac{N_C}{k|P_H|}\mid G)\ge\frac{c'\frac{m}{k}(1-\theta_T)^k}{ckm}=\frac{c'(1-\theta_T)^k}{k^2c}=\Omega(\frac{1}{k^2}n^{-C\alpha})$. Thus, $\mathbb{E}(R)\le 1-\Omega(\frac{1}{k^2}n^{-C\alpha})+\frac{6}{n}$

Note that since $m=\Omega(n^{2C\alpha+\epsilon})$, we have $m^{-1/2}=O(n^{-C\alpha-\epsilon/2})=o(n^{-C\alpha})$, and so $\mathbb{E}(R\,|\,G)\le 1-\Omega(\frac{n^{-C\alpha}}{k^2})=1-\omega(\frac{1}{\sqrt{m}})$. From this, we get that $\mathbb{E}(R)\le 1-\omega(\frac{1}{\sqrt{m}})$. Thus, if we do not handle clipping anchors explicitly in the definition of recoverability, the expected recoverability converges more slowly than $1-O(\frac{1}{\sqrt{m}})$.

Below we prove the statements used in the informal analysis above. Recall that $G=EC\wedge F1\wedge F2$

Lemma D.1 (Working in ***G***) The expected number of clipping anchors is at least $\Omega(mk(1-\theta_T)^k)$

Proof Partition $S[p+1:p+m']$ into $\frac{m'}{k}$ regions of length k. For each k-mer, there can be a clipping anchor produced by a simple 'kink', i.e. an insertion at position i of $S[i]$ followed by a deletion of $S[i]$. If there are two 'kinks', the result is an anchor since the substrings on S and S' are left unchanged and that anchor clearly clips P_H due to the kinks.

There are $\binom{k}{2}$ ways to select the locations for the two 'kinks' and the probability that they occur is $(\theta_i\theta_d(1-\rho_i')\frac{1}{\sigma})^2(1-\theta_T)^{k-2}=c(1-\theta_T)^k$ after absorbing constants and writing $(1-\theta_T)^{k-2}=\frac{(1-\theta_T)^k}{(1-\theta_T)^2}$.

Thus, the expected number of clipping anchors is at least $c\frac{m'}{k}\binom{k}{2}(1-\theta_T)^k=\Omega(m'k(1-\theta_T)^k)$ after summing over all $\frac{m'}{k}$ k-mers in $S[p+1:p+m']$. Using the fact that $m'=cm$ under G gives the result. □

Lemma D.2 (Working in ***G***) The expected number of homologous anchors is at most $O(m(1-\theta_T)^k)$.

Proof For a homologous anchor to occur, there must be a sequence of at least $k-1$ positions in $S[p+1:p+m']$ for which there are no mutations at all. This occurs with probability at most $(1-\theta_T)^{k-1}$. An overestimate gives that every point in $S[p+1:p+m']$ contributes a homologous anchor, showing that $\mathbb{E}(N_H)\le m'(1-\theta_T)^{k-1}=\frac{m'}{1-\theta_T}(1-\theta_T)^k=O(m(1-\theta_T)^k)$ under G. □

Since $mk(1-\theta_T)^k\gg m(1-\theta_T)^k$, as $n\to\infty$, clipping anchors are, in fact, the dominant type of anchor.

I. Recoverability convergence under varying insertion lengths

Fig. I.1 shows log-log plots of the average recoverability error against the average length of S' for $\theta_T=0.159$ and a range of γ values: $\gamma\in\{0.05,0.25,0.50,0.75,0.95\}$. Recall that γ governs the average insertion length, with larger γ corresponding to larger insertion lengths. The slope for each of these fits is at most -0.50, providing evidence that $\mathbb{E}(R)=1-O(\frac{1}{\sqrt{m}})$ is independent of the value of γ (provided that $\gamma<1$) as claimed in the theory.

J. Additional INDELible and PBSIM3 Experiments

The experiments presented in the main paper stress test the parameter regime used in our theoretical results assuming the mutation model from Definition 1. In particular, mutation types (substitutions, insertions, deletions) occurred independently at each position in the reference string, insertions followed a geometric distribution, and deletions followed a Bernoulli distribution. These assumptions are mathematically convenient but simplified compared to real genomic mutation processes.

To address this, we ran simulations with PBSIM3 and INDELible, and calculated the expected error scaling in the log-log plot for recoverability and the predicted runtime. Figure J.1 shows the results. Below, we present a brief discussion of the results, and what they show.

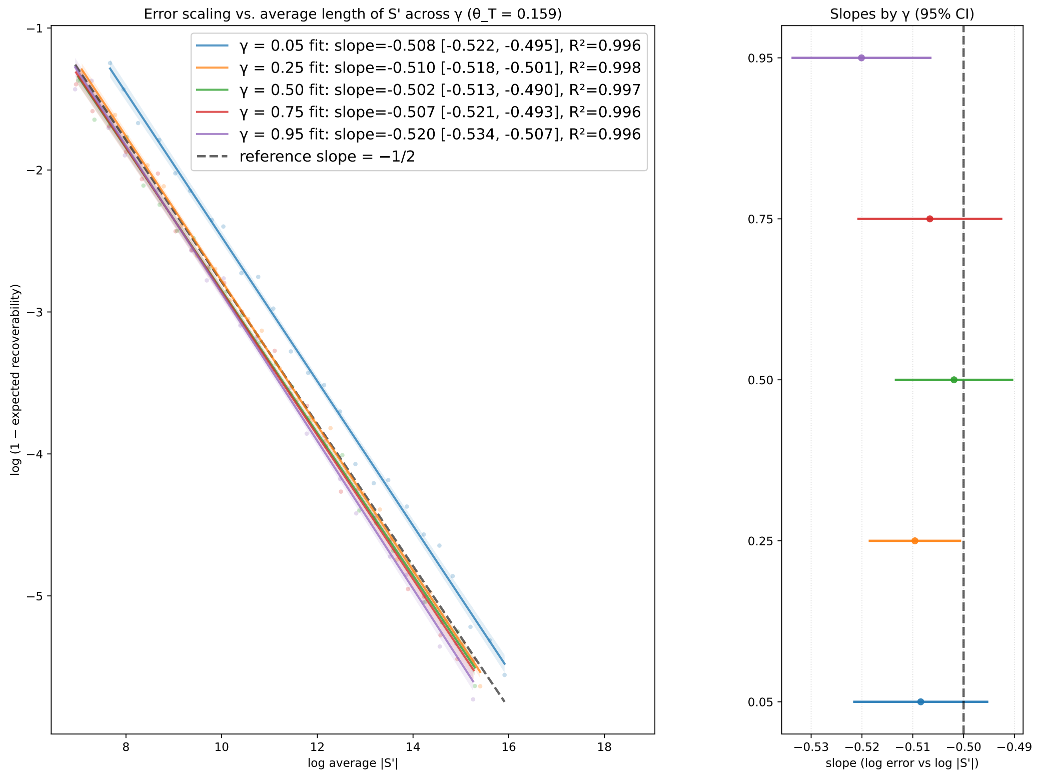

Fig. I.1. Left: log–log fit of the average recoverability error against the average length of S' for $\gamma \in \{0.05, 0.25, 0.50, 0.75, 0.95\}$, and $\theta_T = 0.159$. Right: slopes (95% CIs) with reference line at -0.50.

In the first experiment, we use INDELible (Fletcher and Yang 2009). Although typically used to test phylogenetic inference methods, INDELible also simulates mutations realistically. We use INDELible with the Hasegawa-Kishino-Yano (HKY) substitution model (Hasegawa *et al.* 1985), indel power-law length distribution with exponent 1.8 and maximum of 100 occurring at rate 0.05 relative to the substitution rate, a branch (divergence) length of 0.10, and a simple two node (root → tip) tree. Root sequences are drawn from human chromosome 1 (hg38). As before, the exact homology is recoverable. Figure J.1 shows that the theoretical recoverability predictions from before hold in this regime: the empirical expected error scales as $m^{-0.559}$. The theoretical runtime scaling also holds and, in fact, the empirical runtime grows much more slowly than predicted.

The second experiment uses PBSIM3, a long-reads simulator that introduces realistic errors. Given a substring S of human chromosome 1 (hg38), we run PBSIM3 on S to get a noisy 'read' S' mimicking what one would obtain from PacBio RSII, retrieve the true alignment between the reference and the read, and run seed-chain-extend on the pair (S, S'). This experiment realistically mimics how seed-chain-extend is used in practice, to align reads to a reference. Note that we treat the true alignment in this experiment as the true homology between S and the 'mutated' substring S'. Note that the empirical expected recoverability converges to 1 slightly slower than the predicted theory; the average empirical error scales as $m^{-0.425}$ rather than $m^{-1/2}$; however, the 95% CI contains the error scaling as $m^{-1/2}$. As in the INDELible experiment, the runtime predictions from the theory hold.

Note that, unlike the experiments in the main paper, there is no total mutation rate in either INDELible or PBSIM3. To make analogous comparisons, we empirically estimated the effective total mutation rates for our experimental configurations and used those in the n, m, k scaling schedule.

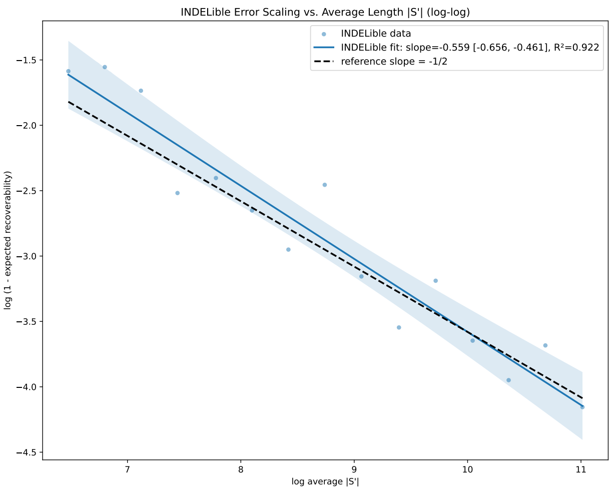

(a) We run INDELible with the HKY substitution model, indel power-law length distribution with exponent 1.8 and maximum of 100. The plot above shows that the empirical recoverability error scales like $|S'|^{-0.55}$, which is consistent with the theory.

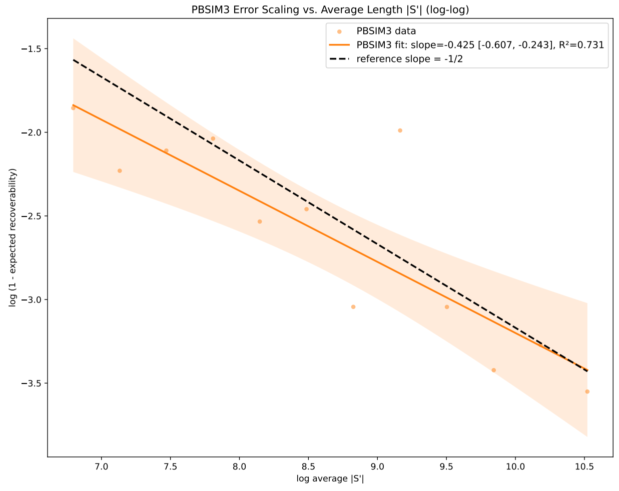

(b) Using PBSIM3 to generate simulated long-reads with errors from a reference S drawn from human chromosome 1 (hg38), obtain S' (the noisy full read), and run seed-chain-extend on the pair (S,S'). The average empirical recoverability error scales like $|S'|^{-0.425}$, slightly worse than predicted, though $|S'|^{-1/2}$ is still in the 95% CI.

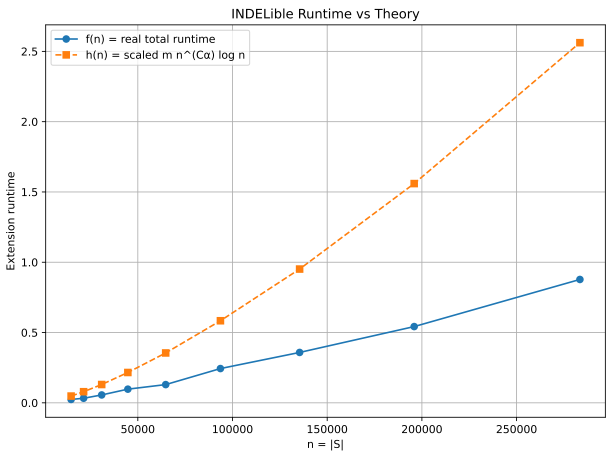

(c) We track the average total empirical runtime for seed-chain-extend in the INDELible experiment. The average empirical runtime is well below the theoretically predicted runtime.

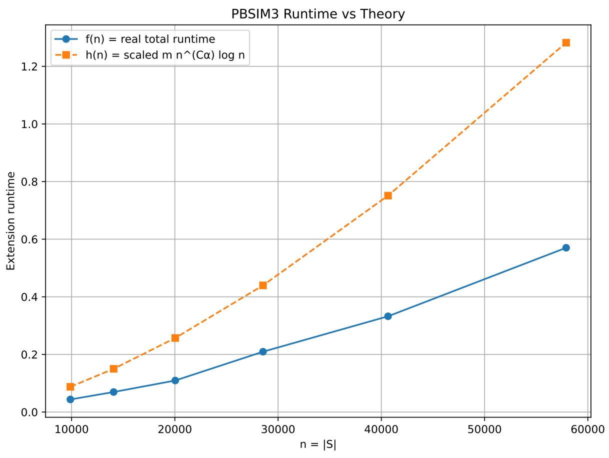

(d) Similarly, we track the average empirical runtime for seed-chain-extend in the PBSIM3 experiment. It is consistent with the theoretical predictions.

Fig. J.1. Theoretical versus empirical recoverability and runtime results for seed-chain-extend in the INDELible and PBSIM3 experiments.
